# Multicohort analysis of the maternal age effect on recombination

**DOI:** 10.1038/ncomms8846

**Published:** 2015-08-05

**Authors:** Hilary C. Martin, Ryan Christ, Julie G. Hussin, Jared O’Connell, Scott Gordon, Hamdi Mbarek, Jouke-Jan Hottenga, Kerrie McAloney, Gonnecke Willemsen, Paolo Gasparini, Nicola Pirastu, Grant W. Montgomery, Pau Navarro, Nicole Soranzo, Daniela Toniolo, Veronique Vitart, James F. Wilson, Jonathan Marchini, Dorret I. Boomsma, Nicholas G. Martin, Peter Donnelly

**Affiliations:** 1 Wellcome Trust Centre for Human Genetics, University of Oxford, Oxford OX17BN, UK; 2 Illumina, Inc., Chesterford Research Park, Little Chesterford CB10 1XL, UK; 3 QIMR Berghofer Medical Research Institute, Brisbane, Queensland 4006, Australia; 4 Department for Biological Psychiatry, Vrije Universiteit, Amsterdam 1081, The Netherlands; 5 Institute for Maternal and Child Health—IRCCS Burlo Garofolo, University of Trieste, Trieste 34137, Italy; 6 MRC Human Genetics Unit, MRC Institute of Genetics and Molecular Medicine, University of Edinburgh, Edinburgh EH4 2XU, UK; 7 Wellcome Trust Sanger Institute, Hinxton CB10 1HH, UK; 8 Division of Genetics and Cell Biology, San Raffaele Scientific Institute, Milan 20132, Italy; 9 Institute for Population Health Sciences and Informatics, University of Edinburgh, Edinburgh EH16 4TJ, UK; 10 Department of Statistics, University of Oxford, Oxford OX1 3TG, UK

## Abstract

Several studies have reported that the number of crossovers increases with maternal age in humans, but others have found the opposite. Resolving the true effect has implications for understanding the maternal age effect on aneuploidies. Here, we revisit this question in the largest sample to date using single nucleotide polymorphism (SNP)-chip data, comprising over 6,000 meioses from nine cohorts. We develop and fit a hierarchical model to allow for differences between cohorts and between mothers. We estimate that over 10 years, the expected number of maternal crossovers increases by 2.1% (95% credible interval (0.98%, 3.3%)). Our results are not consistent with the larger positive and negative effects previously reported in smaller cohorts. We see heterogeneity between cohorts that is likely due to chance effects in smaller samples, or possibly to confounders, emphasizing that care should be taken when interpreting results from any specific cohort about the effect of maternal age on recombination.

## Abstract (web summary)

The question of whether recombination rate increases with maternal age is controversial, with conflicting prior evidence. Here, Martin *et al.* analyse nine cohorts in the largest SNP-based analysis of this question and find a small positive increase with maternal age in the number of crossovers.

## Graphical abstract

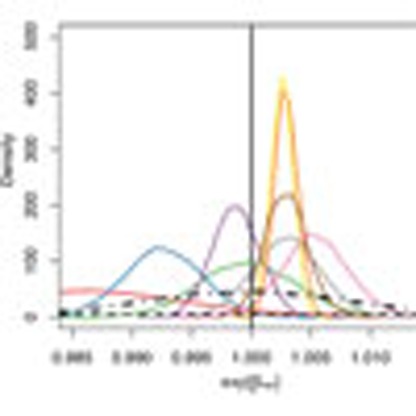

Failure of recombination is known to contribute to human aneuploidies by producing nondisjunction of homologous chromosomes during meiosis I (reviewed in refs 1, 2). Aneuploidies are the leading genetic cause of miscarriage and developmental disabilities in humans, and the vast majority are of maternal origin^3^. The frequency of aneuploidies increases dramatically in older mothers^4^: only about 2% of clinically-recognized pregnancies in women under 25 are trisomic, compared with 35% in women over 40 (ref. 1).

It has been proposed that changes in recombination rate in normal oocytes are responsible for the maternal age effect on aneuploidies^5^. To test this directly, one would need to examine recombination levels in oocytes taken from the same women at multiple time points. However, obtaining such samples is difficult, so several groups have instead used pedigree studies to examine the effect of maternal age on the number of crossovers in liveborn offspring^6^,^7^,^8^,^9^. These studies have yielded conflicting results.

Kong *et al.*^6^ analysed about 1,000 microsatellite markers in 14,140 maternal meioses from Iceland, and used data on age rounded to the nearest 5 years. They fitted a linear mixed model that accounted for differences in baseline rates between mothers to estimate the expected change in the number of crossovers per year of maternal age, *β*_age_. They reported that *β*_age_=0.082 (s.e.=0.012, *P*<10^−8^) but noted that the relationship between recombination rate and maternal age was not linear in their data, and that there was a large jump from age 30–35. They hypothesized that the apparent increase in the number of crossovers with age was due to a selection effect, such that eggs with more crossovers were more likely to give rise to a live birth. Thus, they predicted that women with higher recombination rates would have more children, and indeed, they found that the number of crossovers was positively associated with family size (+0.0109 children per crossover, s.e. 0.0041, *P*=0.0076). It is for this reason that it is important to account for the baseline recombination rate of the mother when analysing the maternal age effect, since the women who have children later might simply have higher rates throughout their lives.

In the first study of recombination using genome-wide SNP data, Coop *et al.*^7^ analysed Affymetrix 500K chip data from 725 Hutterite individuals, part of a large 13-generation pedigree. They found an increase of 0.19 crossovers per year using crossover counts and ages adjusted by the mean for the parent (s.e. 0.093, *P*=0.035 by permutation). Like Kong *et al.*^6^, they found a more pronounced increase in older women: mothers over 35 had an average of 3.1 more crossovers than those under 35 (*P*=0.028 using a within-family permutation test). Coop *et al.* also found that the mean maternal recombination rate was significantly correlated with family size (Spearman *ρ*=0.24, *P*=0.027).

Two more recent pedigree studies^8^,^9^ found that the recombination rate decreases with maternal age in humans, as it does in mice^10^. Hussin *et al.*^8^ analysed 195 maternal meioses from a French Canadian cohort. Using a linear mixed model with the mother as a random effect, they estimated that *β*_age_=−0.49 crossovers per year (*P*=0.0017), and they obtained similar results using adjusted counts within families (*β*_age_=−0.42, *P*=0.0047). Bleazard *et al.*^9^ analysed 338 meioses from Mongolian and Korean families. They regressed maternal crossover count on maternal age, including mother as a factor, and found *β*_age_=−0.29 crossovers per year (*P*=0.03).

Recently, Campbell *et al.*^11^ reported an analysis of recombination in about 4,200 families from the 23andMe data set in which they found an increase of 0.067 maternal crossovers per year. However, as they noted, this analysis could be confounded if mothers with higher recombination rates tend to have more children later in life, since they fitted a simple linear regression that did not account for the baseline rate of the mother. When Campbell *et al.* restricted the analysis to 2,184 phase known meioses (from families with more than two children) and ran the regression on the adjusted counts, they did not find a significant association with age (*P*=0.11).

Thus, there is a lack of consensus about the size and direction of effect of maternal age on recombination rate.

There are likely several factors contributing to the conflicting results between different cohorts in the previous studies^6^,^7^,^8^,^9^. There was considerable heterogeneity in the number of genetic markers used, in genotype data filtering and quality control, in the accuracy of the age data available, and in the methods used to call crossovers and to test the age effect. Here, we revisit this question in nine different cohorts (Table 1; Supplementary Table 1). These include the French Canadians in which Hussin *et al.* had previously investigated the maternal age effect, six cohorts that O’Connell *et al.* had used in a study on phasing in populations with high levels of relatedness^12^, and two cohorts from twin registries not previously studied for this purpose. We attempt to eliminate some of the possible confounders by applying the same methodology, as far as possible, to all cohorts, to see whether applying a standardized approach makes the results more consistent. We find that the maternal age effect on the number of crossovers is small and positive, and that the differences between cohorts are likely due to chance effects in small samples.

## Results

### Calling crossovers

We analysed the maternal age effect on recombination in the largest sample to date using SNP chip data (Table 1; Supplementary Table 1). We applied two methods to call crossovers; these use different approaches to phase the parents and to account for genotyping errors that can look like recombination events over short intervals. NFTOOLS^8^, based on the method by Coop *et al.*^7^, phases the parental chromosomes using patterns of transmission to the children, and then identifies positions where a child’s chromosome switches from copying one parental haplotype to the other. It requires both parents and at least three children (‘informative nuclear families’) to assign crossovers to children. In contrast, the Hidden Markov Model (HMM) method, duoHMM^12^, phases all samples in the cohort as though they are unrelated, and then incorporates family information to correct phasing, detect genotyping errors and call crossovers. It requires only a duo (parent–child pair) to call crossovers, but as O’Connell *et al.* showed^12^, it has greatest power for duos from informative nuclear families or families in which the grandparents are also genotyped. We refer to duos/meioses from such families as ‘fully informative’, and all other duos/meioses as ‘partially informative’ (termed ‘uninformative’ in ref. 12).

The number of crossovers called by NFTOOLS agreed well with expectation (Supplementary Fig. 1), but duoHMM seemed to be over-calling for several of the cohorts, notably those from more outbred populations with low background relatedness (Queensland Twin Registry (QTR), Netherlands Twin Registry (NTR) and French Canadians (FC)) (Supplementary Fig. 2; Supplementary Table 2). We suspected this was due to phasing errors, since we would expect the phasing to be better in cohorts with more low-level relatedness. We found that removing double crossovers called within short intervals (see Methods), which were probably due to switch or genotype errors, brought the number in line with expectation for all cohorts (Supplementary Fig. 2). All duoHMM results described below were based on these filtered calls, but we also obtained very similar results using the raw calls (compare Supplementary Table 3 with Table 2). There was good agreement between the final NFTOOLS and duoHMM callsets on the same meioses (that is, from informative nuclear families; Supplementary Fig. 3), with 96% of crossovers overlapping. We estimated that 70–76% of maternal crossovers occurred in HapMap II recombination hotspots, after correcting for the number expected by chance (Supplementary Table 4). This is similar to previous reports in non-African cohorts^8^,^9^,^13^.

### Linear mixed models and meta-analysis

**Figure 1:**
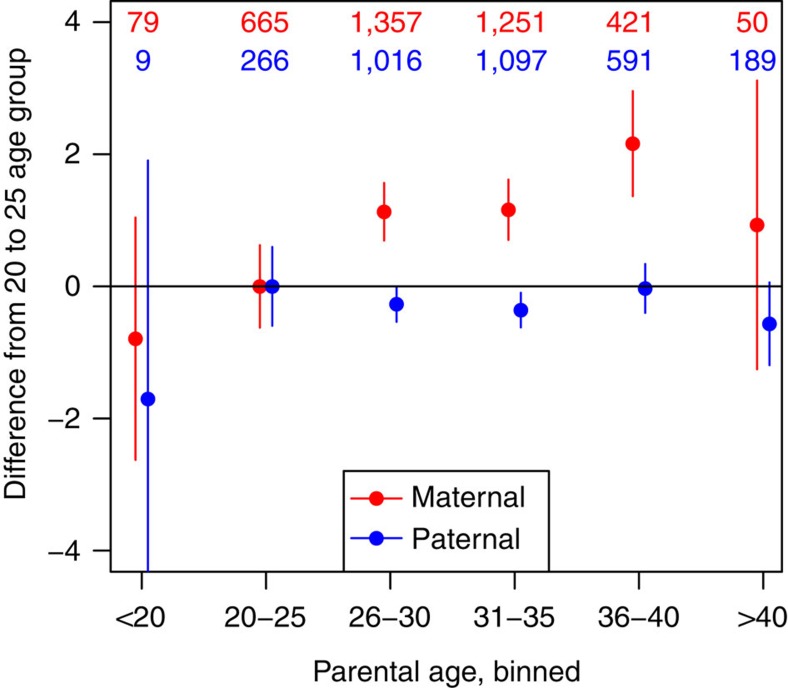
Plot of the number of crossovers as a function of binned parental age. Ages are grouped into 5-year bins, relative to parents of between 20 and 25 years old. The number of meioses in each age bin is indicated along the top, in red for maternal and blue for paternal. Note that age data were not always available for both parents, hence the total sample size differs for maternal and paternal meioses. Points show means and error bars 95% confidence intervals. This plot is based on data from fully informative duos analysed with duoHMM.

**Figure 2:**
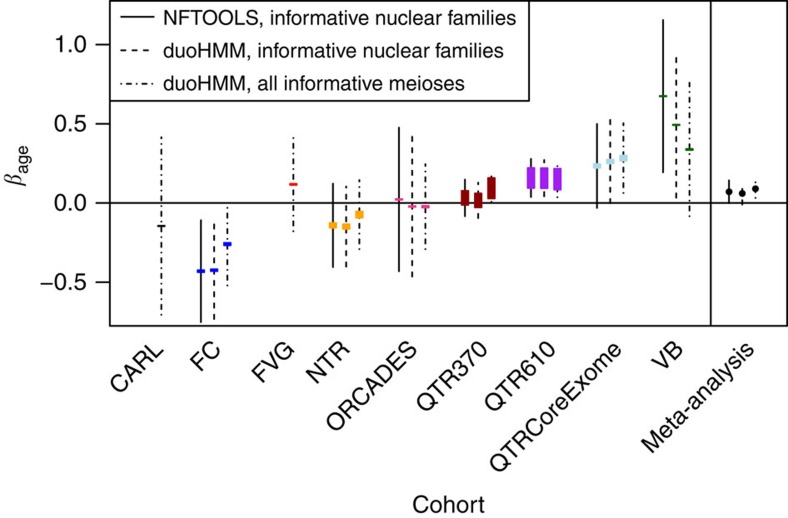
Estimates of the maternal age effect from linear mixed models. The length of the coloured boxes is proportional to sample size. The black lines show 95% confidence intervals, and the line type indicates which data set was used. On the far right are estimates from the meta-analysis, indicated as points with their confidence intervals. The sample sizes for each cohort are listed in the second last column of Table 1.

Exploratory analyses of fully informative meioses suggested a small increase in the number of crossovers with maternal age (Fig. 1), with some suggestion of a larger increase in mothers over 35, as described by Coop *et al.*^7^ and Kong *et al.*^6^, although not in mothers over 39, as described by Campbell *et al.*^11^. However, this simple analysis does not account for differences in baseline recombination rates between mothers. Thus, for each cohort separately, we fitted a linear mixed model to the duoHMM counts from fully informative meioses, treating mothers as a random effect (Supplementary Table 5), following refs 6 and 8. Estimates of the effect of maternal age on the number of crossovers, *β*_age_, varied between −0.26 for the FC cohort and 0.34 for Val Borbera (VB) (Fig. 2; Supplementary Table 5), but only the three QTR cohorts were significantly different from 0 at the 0.05 level (QTR370: *β*_age_=0.09, *P*=0.03, QTR610: *β*_age_=0.15, *P*=0.01, QTRCoreExome: *β*_age_=0.28, *P*=0.01; *P*-values are from a *t*-test in the linear mixed model).

Combining the estimates from all cohorts in a fixed-effects meta-analysis, we found that the effect of maternal age was small but significant (*β*_age_=0.09, *P*=0.002; *Z*-test) (Supplementary Table 6). Similar results were obtained using only informative nuclear families with both NFTOOLS and duoHMM data (Fig. 2; Supplementary Tables 5 and 6), but the effects for FC and VB were more extreme (*β*_*age*_=0.49, *P*=0.049 for VB; *β*_*age*_=−0.42, *P*=0.008 for FC, using duoHMM counts). We suspect that these estimates were more affected by outliers, given the smaller sample size imposed by restricting analysis to informative nuclear families (Supplementary Fig. 4), but we discuss this further below. In the meta-analysis, there was significant heterogeneity in the estimated effects between cohorts, as assessed by Cochran’s *Q* test (Supplementary Table 6).

### Bayesian analysis of the maternal age effect

Hierarchical models that share information between cohorts, while allowing for differences between them, may add power to discern an effect, and have a more natural interpretation. We fitted a variety of such models in a Bayesian framework (see Methods). We found very similar results for NFTOOLS and duoHMM counts on the same set of meioses from informative nuclear families (Supplementary Table 7; Supplementary Fig. 5). Thus, we focus on the duoHMM results below, because this method also allows us to include meioses from all families, not just informative nuclear families, and hence to substantially increase the number studied.

**Figure 3:**
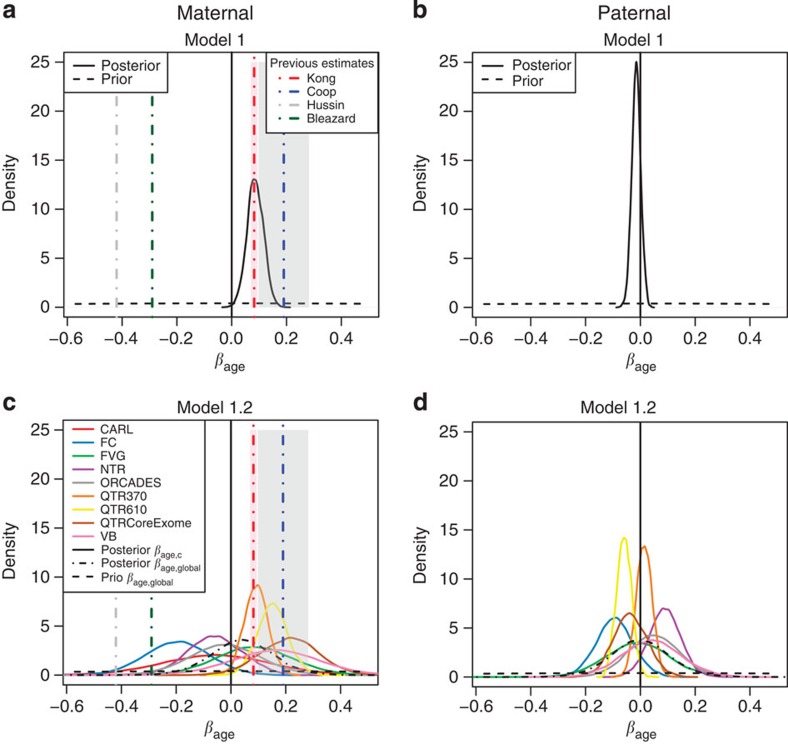
Bayesian posteriors for the age effect from normal models fitted to fully informative meioses. These plots show the priors (dashed lines) and posteriors (solid lines) for *β*_age_ from a normal model fitted to the number of crossovers called in fully informative duos by duoHMM, with either the same (panels **a**,**b**) or different (panels **c**,**d**) age effects for each cohort (see Models 1 and 1.2 in Methods). In the bottom plots, the posterior for *β*_age, global_ is also indicated. The vertical lines show the estimates from previous studies, and the shaded boxes the corresponding standard errors, if reported. Under this model, the expected number of crossovers increases by *β*_age_ per year.

We began by analysing only the fully informative meioses (*n*=3,823 maternal meioses from 1,132 families; 3,558 paternal meioses from 1,073 families). Figure 3 shows the posterior distribution of the age effect, *β*_age_, under a normal model for the number of crossovers in which the mother (or father) effects for parent *j* in cohort *c*, *α*_*j*,*c*_, are drawn from a normal distribution with cohort-specific mean *μ*_*c*_ and variance 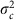, with the cohort-specific means and variances being independently sampled from a common distribution across cohorts. Posterior distributions for the other parameters are shown in Supplementary Fig. 6. We fitted either a common *β*_age_ for all cohorts (Model 1; Fig. 3 top), or a different *β*_age,*c*_ for each cohort *c*, drawn from the same normal distribution with mean *β*_age, global_ and variance 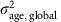 (Model 1.2; Fig. 3 bottom). Our results for Model 1 fitted to maternal crossovers (Fig. 3a) strongly support a small positive effect (Table 2): the 95% credible interval was (0.0267, 0.1451), which is consistent with the effect found by Kong *et al.*^6^ using microsatellite markers, and 99.8% of the posterior distribution for *β*_age_ was concentrated on positive values. Our results are not consistent with the larger positive effect reported in the Hutterites by Coop *et al.*^7^ or the negative effects reported in the original analysis of the French Canadians by Hussin *et al.*^8^ or in the Asian cohort by Bleazard *et al.*^9^.

**Figure 4 f4:**
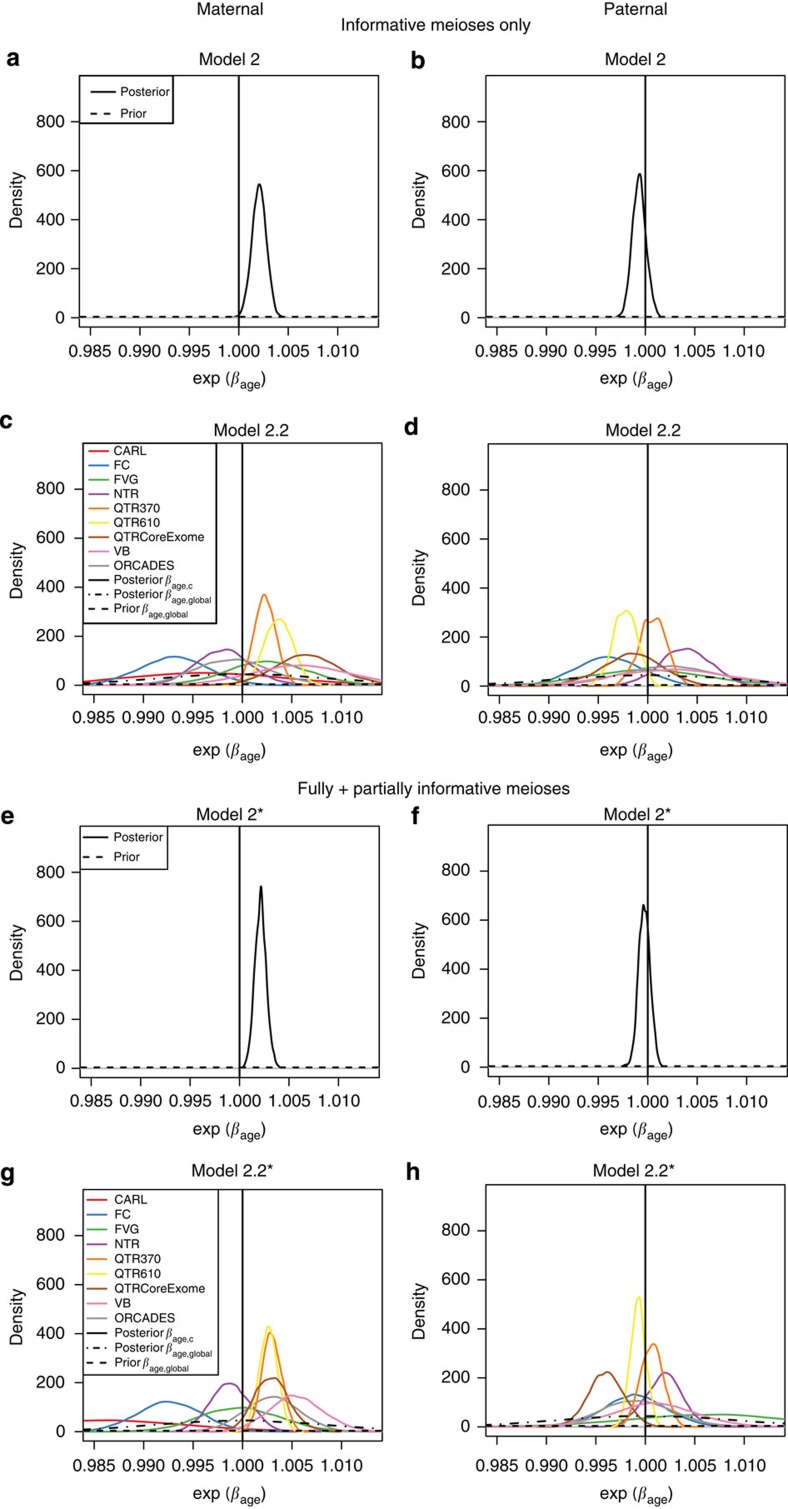
Bayesian posteriors for the age effect from negative binomial models. These plots show the priors and posteriors for 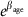 from a negative binomial model fitted to the number of crossovers called by duoHMM in either informative duos only (**a**–**d**) or both informative and partially informative duos (**e**–**h**), with either the same (Models 2 and 2*) or different (Models 2.2 and 2.2*) age effects for each cohort (see Methods). We have plotted 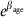 since, under the negative binomial model, the expected number of crossovers increases by this factor per year. The axes have been chosen to facilitate comparison with the normal model shown in Figure 3, assuming a baseline of 38, that is, the bounds [0.985,1.013] on 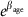 under the negative binomial model are approximately equivalent to the bounds [38 × log(0.985), 38 × log(1.013)]=[−0.57,0.49] on *β*_age_ under the normal model.

When we fitted a more general normal model with cohort-specific age effects (Model 1.2) to maternal meioses, there was some evidence for heterogeneity of effects, with FC showing the strongest negative effect (Pr(*β*_age,FC_<0)=0.95), and QTR610 the strongest positive (Pr(*β*_age, QTR610_>0)=0.98; Fig. 3c). Since it seemed more appropriate for count data, we also fitted a negative binomial model with a common age effect, in which the number of crossovers increased multiplicatively by a factor of 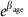 per year (Model 2). The evidence in favour of a positive effect was very similar to that under the normal model (Fig. 4a–d; Supplementary Fig. 7): 99.96% of the posterior distribution for *β*_age_ was concentrated on positive values (Table 2).

The results presented thus far have all been based on data from 3,823 fully informative maternal meioses from 1,132 families. However, duoHMM can call crossovers in any duo, since it uses all individuals in the cohort for phasing. Its performance depends on the amount of low-level relatedness in the cohort, which affects phasing accuracy. Supplementary Figure 8 shows that duoHMM is under-calling crossovers in meioses that are only partially informative, but to a different extent in each cohort, consistent with the results of O’Connell *et al.*^12^. We adapted the negative binomial model to allow for this effect by instead drawing the mother (or father) effects, *α*_*j*,*c*,*f*_, from a normal distribution with mean *μ*_*c*,*f*_ and variance 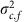 corresponding to the family configuration *f* in cohort c (see Methods for a description of Model 2* and the family configurations). We fitted this to the 6,011 maternal meioses from families with at least two children, requiring multiple children so that the mother effect could be estimated. With this increased sample size, the results were broadly similar (Fig. 4e–h; Supplementary Fig. 9), though the posterior on *β*_age_ (or *β*_age,*c*_) was tighter than when using only the fully informative meioses (compare top four with bottom four plots in Fig. 4).

We emphasize that the interpretation of *β*_age_ is different for the negative binomial models versus the normal model. In the normal model (Model 1), the number of crossovers is expected to increase linearly from the baseline by *β*_age_ per year, whereas in the negative binomial models (Models 2 and 2*), it increases by a factor of 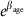 per year. In practice, because *β*_age_ is so small, this makes very little difference. For our estimated *β*_age_ values, the multiplicative 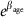-fold increase is close to a linear increase by (*β*_age_ × baseline) per year, at least over a relatively small number of years.

To check that our results were robust to the choice of priors, we fitted Model 2* with an uninformative prior placed in turn on each parameter (see Methods). Our inference about *β*_age_ was not substantially changed: in each model, at least 99% of the posterior density was concentrated on positive values (Supplementary Fig. 10).

All previous studies had found no significant effect of the father’s age at birth on the number of paternal crossovers^6^,^7^,^8^,^9^,^11^. Our results are consistent with this (Table 2; Figs 3 and 4; Supplementary Figures 5, 6, 7 and 9), but it is notable that we see some heterogeneity of estimated paternal age effects between cohorts. For example, for Model 1.2 (Fig. 3d), we find Pr(*β*_age_>0)=0.94 for NTR and 0.09 for FC. We discuss possible explanations for and implications of this heterogeneity later.

### Posterior predictive checks

We conducted posterior predictive checks to assess how well these hierarchical models fitted the data, by comparing the distribution of the observed crossover counts to data simulated under a model with the estimated parameters (Supplementary Figs 11–13). The median number of crossovers was similar between the observed and simulated data for all models. The minimum tended to be too low in data simulated under the normal model (Model 1), which is expected given that it has no lower bound. There was an asymmetry in the distribution of the observed data (Supplementary Fig. 11, red histograms), and, for the fully informative meioses, the negative binomial model (Model 2) captured this better than the normal model (Model 1) (Supplementary Fig. 13).

## Discussion

We have investigated the maternal age effect on recombination in data from over 6,000 meioses from nine cohorts. This is by far the largest SNP-based sample analysed for this question. Kong *et al.*^6^ studied a much larger sample of over 14,000 meioses, using 1,000 microsatellite markers, and had age data rounded to the nearest 5 years.

To estimate the age effect we first applied the same statistical approach as in refs 6 and 8, namely a linear mixed model (Fig. 2; Supplementary Table 5). We found significant effects in some cohorts (positive in VB and the QTR cohorts, negative in FC), and a meta-analysis of all cohorts combined showed a small and significant positive effect (Supplementary Table 6).

In addition to the approach of earlier authors, we also fitted various hierarchical models that accounted for differences between mothers and cohorts, fitting these in a Bayesian statistical framework. Model 2* allowed us to account for the uncalled crossovers in partially informative meioses, so we could increase our sample size by including these. These hierarchical models had good frequentist properties, including low-type I error, and we had good power to detect a multiplicative effect greater than about 1.0015 crossovers per year with the informative meioses (Supplementary Fig. 14).

Our results on 3,823 fully informative meioses (posterior mean for *β*_age_=0.0862 crossovers per year) were very similar to the small positive maternal age effect reported by Kong *et al.*^6^ (0.082 crossovers per year). They are also in line with the estimate reported recently by Campbell *et al.*^11^ (*β*_age_=0.067 crossovers per year), although we note that this effect became nonsignificant when they restricted the analysis to the families in which crossovers could be assigned to children (2,184 meioses). We could reject there being no effect with high probability: 99.96% of the posterior density was concentrated on positive values (Model 2 fitted to informative meioses). When we included partially informative meioses (Model 2*), increasing the sample size to 6,011 meioses, this gave a tighter posterior on the age effect, and we estimated, using the posterior mean of *β*_age_, that the expected number of crossovers increases by 2% over 10 years (95% credible interval (1.1%, 3.0%)). Translation between parameters in this multiplicative model and the additive model we and others have used for fully informative meioses is not immediate. Nonetheless, assuming a baseline number of crossovers of 38, as inferred from the data (Supplementary Fig. 6), shows the multiplicative parameter estimate to be in good agreement with the additive change of ∼0.08 crossovers per year that we and Kong *et al.*^6^ inferred.

Following previous studies^7^,^8^, we removed double crossovers over short intervals since we thought these were likely to be due to genotyping or, for duoHMM, switch errors. This brought the distribution of the number of crossovers closer to expectation for the QTR, NTR and FC cohorts. If, however, some of these double crossover events were real, we would be underestimating the number of crossovers, and if their frequency changed with age, as might be expected given the recent report by Campbell *et al.*^11^, this could be a confounding factor in our analysis. We thus fitted our hierarchical models again using the raw duoHMM data. The 95% credible intervals for *β*_age_ were very similar to those obtained using the filtered data (compare Supplementary Table 3 with Table 2) and we therefore concluded that the filtering did not greatly affect our results.

Although we applied the same methods to all cohorts, we still saw substantial heterogeneity between cohorts in the magnitude and direction of the maternal age effect. There were differences between cohorts in the number of SNPs (Supplementary Table 1), but although this does affect *μ*_*c*_, the mean of the mother effects for cohort c (Supplementary Figs 6, 7 and 9), it is unlikely to affect the estimate of *β*_age,*c*_, since, within a cohort, there is no association between maternal age at birth and the number of SNPs. There could also be inter-cohort differences in family size, which has previously been found to be associated with recombination rate^6^,^7^. If women with higher baseline recombination rates have more children later in life, the number of recombinations will appear to be higher in older mothers. We could not explicitly include family size as a covariate in our analyses, since we did not have that information, but we should, in theory, be capturing this effect by including the mother effect, *α*, in our model. Since the *α* for each mother is drawn from the same distribution as the other mothers in the cohort, we might expect some shrinkage towards the mean mother effect. This could potentially, for example, reduce the estimate of *α* for high-recombining mothers and thus inflate *β*_age,*c*_. However, it is not clear how such a phenomenon could cause the negative *β*_age,*c*_ estimates in some cohorts, notably in the French Canadians. Notably, we still see heterogeneity when fitting the linear mixed model to each cohort separately (Fig. 2), in which the mother effects are independent of one another, so we do not think differences in family size can account for this.

There is information about the levels of between-cohort heterogeneity expected under the null from analyses of paternal meioses. All previous studies (and our analyses) have suggested that there is no paternal age effect on numbers of crossovers. Further, the explanations proposed for the maternal age effect on aneuploidies (the production line model^5^, or loss of cohesin during the long meiotic arrest^14^) are not relevant to males. There is clearly still some heterogeneity between cohorts for paternal age effect estimates (Figs 3d, 4d-4h, [Fig f4]), which must just reflect variation under the null hypothesis.

We observed that the number of crossovers was more over-dispersed in females than in males, relative to the mean: the posteriors for *ω*, the over-dispersion parameter, put more weight on smaller values for males versus females in Supplementary Figs 7 and 9. We should thus expect more variation, and less precise estimation, in females than in males, and indeed this is the case in our data: the posteriors for *β*_age_ are tighter for fathers than for mothers (for example, compare Fig 3c–d). Taken together, this suggests that the differences in *β*_age_ we see between cohorts may simply reflect chance effects. We note that the large negative effect reported by Hussin *et al.*^8^ in informative nuclear families from the French Canadians was attenuated when the sample was expanded from 106 to 158 to include all fully informative meioses (that is, adding families with only two children, but with a third generation; see Supplementary Fig. 4).

It is possible that the heterogeneity in effects between cohorts is due, at least in part, to biological differences or to confounders. We think that true biological differences are unlikely, given that meiotic recombination is such a fundamental process. However, if the relationship between maternal age and recombination rate were nonlinear, and if the cohorts had different maternal age distributions, this might explain why they show varying directions and magnitudes of effect. The age distributions do differ between cohorts (Supplementary Fig. 15), but we saw no evidence for nonlinearity with age in the residuals (Supplementary Fig. 16), so we do not think this a likely explanation.

We can think of two possible confounders that may be contributing to the heterogeneity. The first is the use of assisted reproductive technologies (ART), which might interfere with normal recombination processes (for example, *in vitro* fertilization might select for eggs with more crossovers). These are common in mothers of twins, who make up a substantial fraction of our sample (the QTR and NTR cohorts). About 27 and 10% of the mothers of informative duos in the QTR and the NTR reported that they had some form of ART. We found that stratifying QTR and NTR mothers by ART use in the hierarchical models did not reveal any clear differences in the age effect (Supplementary Fig. 17a), although we had limited power for these smaller sample sizes.

A second possible confounder is oral contraception. This suppresses ovulation, and so, if eggs are released in the order they are produced during fetal development, as happens in mice^15^, the age of eggs released at a certain point in a woman’s lifetime will differ depending on whether and for how long she has taken oral contraception. If there is an association between meiotic entry and recombination, as in the production line model suggested by Henderson and Edwards^5^, oral contraceptive use could confound the measured effect of maternal age on the number of crossovers. We note that a recent study argued that the production line model could not explain the maternal age effect on aneuploidies^16^. However, there is evidence that mothers over 40 who have taken oral contraception for longer have a significantly lower risk of having a fetus with a common trisomy^17^, suggesting that suppressing ovulation could somehow affect the recombination levels of the eggs subsequently released. It is notable that the largest positive estimate of *β*_age_ previously reported was in the Hutterites^7^, most of whom probably do not use any birth control. We had some data on oral contraceptive use for the NTR and ORCADES cohorts. We saw no clear difference in *β*_age_ between those who had taken oral contraception before the birth of their children and those who had not (Supplementary Fig. 17b). Nonetheless, we had reduced power for these smaller samples, and thus, the effects of oral contraception on recombination levels and aneuploidy risk merit further investigations in much larger samples for which more detailed data, for example on the length of oral contraceptive use, are available.

In conclusion, we analysed over 6,000 meioses from across nine cohorts, and found that the maternal age effect on the number of crossovers is small and positive. We can rule out the larger positive and negative effects previously reported, and we do not replicate the larger increase in number of crossovers for mothers over 39 described by Campbell *et al.*^11^ (Fig. 1). Neither our results nor those of other studies apply directly to either maternal recombination rates nor to the maternal age effect on aneuploidies because all studies count crossovers in live births. Since the chance of fetal survival may increase with the amount of recombination, observed effects on numbers of crossovers may not reflect changes in the recombination rate in oocytes. Nonetheless, our results are still of inherent biological interest, and further insights into the recombination process could shed light on factors affecting aneuploidy rates. The positive effect we observe can probably be explained by the selection hypothesis proposed by Kong *et al.*^6^: because the factors that cause aneuploidies increase with maternal age, eggs with more crossovers are more likely to be able to overcome these and give rise to a live birth in older mothers. We see some evidence for heterogeneity between cohorts, and we can exclude differences in crossover-calling methodology or in statistical approaches as explanations for this. It may instead be attributable to hidden confounders, possibly including, but not limited to, assisted reproductive technologies or oral contraception, or simply to chance effects in smaller samples. Thus, our findings also emphasize that care should be taken in interpreting results from any specific cohort.

## Methods

### Cohorts

The cohorts used are detailed in Table 1 (maternal meioses) and Supplementary Table 1 (paternal meioses and genotyping chips used). The QTR and NTR cohorts were collected for a variety of studies on behavioural and medical traits. In both cohorts, all families include one or more pairs of monozygotic and/or dizygotic twins, as well as one or both parents and, in some cases, additional siblings. For the analyses in this paper, one child of each monozygotic pair was removed. Permission was received to use these data for studying recombination from the Central University Research Ethics Committee of the University of Oxford, from the Ethics Committee of the Queensland Institute of Medical Research, and from the Central Ethics Committee on Research Involving Human Subjects of the VU University Medical Centre, Amsterdam. Informed consent was given by study participants.

The French Canadian cohort consists of 478 individuals from 89 overlapping nuclear families. This is the same cohort studied by Hussin *et al.*^8^. The remaining cohorts were studied in O’Connell *et al.*^12^, and are composed of both unrelated individuals and pedigrees of various sizes. These are from isolated populations: the Orkney Islands (ORCADES^18^), the Dalmatian islands of Vis and Korcula off Croatia^19^ (treated separately), and three Italian populations, VB^20^, Friuli Venezia Giulia^21^ and Carlantino^21^.

Data on the use of ART and oral contraception were available for a subset of families in the QTR, NTR and ORCADES cohorts. For the QTR, some mothers had been surveyed during clinic visits, online and/or by phone or postal questionnaire about whether they had ever had hormone treatment, *in vitro* fertilization or alternative or natural fertility treatments. For the NTR, the mothers had been asked whether each child was conceived spontaneously or not (for example, using hormones or *in vitro* fertilization), whether they had ever taken oral contraception, and whether they had taken it before the birth of the twins. For the ORCADES cohort, we had data on whether each mother had ever taken oral contraception. For some analyses, we stratified QTR, NTR and/or ORCADES mothers by ART or oral contraceptive use before the birth of their children.

### Preparation of genotype data

We first ran KING^22^ on each cohort to detect any pedigree errors and find any cryptic first-degree relationships. In addition to the quality control performed on individual cohorts before this project, we also removed SNPs with a minor allele frequency in the founders <5%, a *P*-value from a test of Hardy–Weinberg equilibrium in the founders <0.01, or >1% Mendelian errors. We set all remaining Mendelian errors to missing, as well as any genotypes flagged as unlikely by Merlin’s error detection algorithm^23^. SNPs with >1% missingness were then removed. For the QTR, we split the sample into three sets of families to maximize the intersection of the SNPs genotyped on all family members: QTR610—families in which all individuals had been genotyped on the Illumina Human610 or Human660W chip; QTR370—families in which at least one individual had been genotyped on the Illumina CNV370 chip; QTRCoreExome—families in which all individuals had been genotyped on the HumanCoreExome chip. For ORCADES, individuals were genotyped on either the Illumina HumanHap300 or the Illumina Omni Express chip. Since most families had individuals genotyped on different chips, for all families we used the intersection of the chips across the whole cohort. The final number of SNPs used for all cohorts is shown in Table 1.

### Calling crossovers

There are several different methods^6^,^7^,^8^,^9^ for calling crossovers using genotype data from pedigrees. Essentially, these rely on inferring the transmission patterns of polymorphic markers from parents to their offspring. A key requirement is that they take into account the possibility of genotype errors which can look like recombination events over short intervals. We used two different methods for calling crossovers. The first, NFTOOLS^8^, based on the approach of Coop *et al.*^7^, is a heuristic method that phases the parental chromosomes using patterns of transmission to the children, and then identifies positions where a child’s chromosome switches from copying one parental haplotype to the other. Following Hussin *et al.*^8^, we removed double crossovers within five informative markers of each other, since these were likely due to genotyping errors.

The second method, duoHMM^12^ involves first phasing the samples with SHAPEIT2 (ref. 24) ignoring family information, and then running a HMM on every parent–child duo. This HMM corrects phasing errors inconsistent with the pedigree structure, detects genotyping errors, and calls crossovers, assigning a posterior probability *P* to each. Following O’Connell *et al.*, we kept only the crossovers with *P*>0.5. We found the number of crossovers called at this threshold was somewhat higher than expected in some cohorts (relative to established genetic maps). Hence we applied some further filtering, excluding double crossovers within *X* SNPs of each other, where *X* was the average number of SNPs per megabase for that cohort, given in Supplementary Table 2. We also fitted the hierarchical models described below on the raw counts, to verify that our results were robust to this filtering (compare Table 2 with Supplementary Table 3).

NFTOOLS requires families with both parents and more than two siblings genotyped to assign crossovers to particular children; we refer to these as ‘informative nuclear families’. In contrast, duoHMM can, in theory, call crossovers in any parent–child duo, because it uses all individuals in the cohort to phase the parents rather than relying on having three offspring. However, it has reduced power if there are insufficient first-degree relatives to phase the parents exactly. We refer to duos/meioses from families with both parents and at least three children, or from three-generation pedigrees, as ‘informative’, and all other duos/meioses as ‘partially informative’ (these were termed ‘uninformative’ in ref. 12).

We applied the hierarchical models presented below to either fully informative meioses (Models 1 and 2), or to fully and partially informative meioses (Model 2*), in both cases from families with at least two children. For Model 2*, we use the following six family configurations: families with at least three children and one or both parents (configurations 1 and 2, respectively), families with two children and one or both parents (configurations 3 and 4), and families with two children, the grandparents (on the side of the relevant parent) and one or both parents (configurations 5 and 6). Configurations 2 and 6 are fully informative, and the rest partially informative.

### Exploratory analysis, linear mixed models and meta-analysis

To test the effect of parental age on recombination, we began by examining the mean number of crossovers for parental ages binned into 5-year intervals. The 95% confidence intervals shown in Fig. 2 were calculated assuming the mean number of crossovers followed a normal distribution (as would be expected by the Central Limit Theorem). Next, to test the effect more formally and account for differences in baseline recombination rates between individuals, we followed the approach of Hussin *et al.*^8^ and fitted a linear mixed model on the maternal or paternal crossover counts:




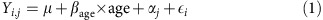




where *Y*_*i*,*j*_ is the number of crossovers for child *i* of parent *j*, *μ* is an intercept, *β*_age_ is the effect of the age of parent *j* at the birth of child *i* (in crossovers per year), *α*_*j*_ is the normally distributed random effect of parent *j*, and 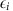 is a normally distributed error term. Hussin *et al.*^8^ included the number of children as a covariate, but since, for most cohorts, we only knew the number of genotyped children, not the total, we could not do this. This model was fitted to the meioses from informative nuclear families for every cohort separately using the nlme package in R (ref. 25). We then carried out a fixed-effect meta-analysis on the *β*_age_ estimates using the metafor package^26^, weighting them by their inverse variances^27^.

### Hierarchical models

We next fitted a variety of hierarchical models to test the maternal age effect. These accounted for differences between families and between cohorts. All models were fitted on the paternal crossovers too, using the priors detailed at the end of this section. The first model was a simple normal model on crossover counts:




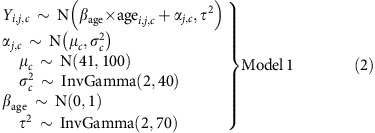




where *Y*_*i*,*j*,*c*_ is the number of maternal crossovers for child *i* of mother *j* from cohort *c*, *α*_*j*,*c*_ is the effect of the mother, *μ*_*c*_ and 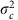 are the mean and variance respectively of the mother effects for cohort *c*, and *τ*^2^ is the residual variance.

We chose conjugate distributions for the priors and hyperpriors, and used an empirical Bayes approach to choose some of the parameters for the prior on the residual variance *τ*^2^ and for the hyperpriors for *μ*_*c*_ and 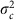. We specified the mean value of the prior for *μ*_*c*_ to match approximately the mean number of crossovers for females (41) reported in Hussin *et al.*^8^, and we set the variance to be large (100) since we found that a tight prior on *μ*_*c*_ influenced the estimates of *β*_*age*_. The 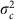 prior was chosen so that its 95% credible interval encompassed the empirical variances in the average number of crossovers for a parent for each cohort. Given that the previous estimates for *β*_age_ had ranged between −0.49 and +0.19, we chose a *N*(0,1) prior for this parameter. We chose the prior for *τ*^2^ so that its mean would be approximately equal to the variance in the number of maternal crossovers across all fully informative meioses.

We also fitted a more general model which allowed for heterogeneity in the age effect between cohorts:




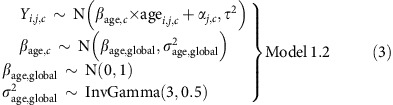




where *β*_age,*c*_ is the age effect for cohort *c*, and *β*_age, global_ and 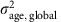 are the mean and variance respectively for the age effects. We used the same hierarchy for *α*_*j*,*c*_ and the same priors for *τ*^2^, *μ*_*c*_ and 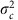 as in Model 1.

Although the normal model has an easy interpretation and seemed to fit the data quite well in the posterior predictive checks, it is not strictly appropriate for count data. Rather than use a Poisson model, which constrains the variance to be equal to the mean, we considered a negative binomial model, using the parameterization:




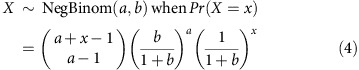




for 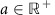, 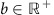 and 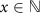. This gives:




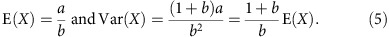




We fitted the following model:




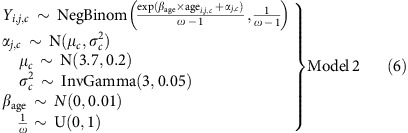




This gives E(*Y*_*i*,*j*_)=exp(*β*_age_ × age_*i*,*j*,*c*_+*α*_*j*,*c*_) and Var(*Y*_*i*,*j*_)=*ω*E(*Y*_*i*,*j*_), where *ω* is the over-dispersion parameter. We put a uniform U(0,1) prior on *ω*^−1^ so that *ω* would be constrained to the range (1, ∞). We used the exponential link function since the shape parameter of the negative binomial distribution, *a*, needs to be strictly positive. We adjusted the priors to account for the transformation of the parameters, so that they would be similar to the priors for Model 1, for example, E(*μ*_*c*_)=log(41)≈3.7.

The models described so far were fitted to data from informative meioses from families with at least two children (with only one child, we cannot estimate *α*_*j*,*c*_), excluding cohorts with fewer than 20 such meioses. Since including partially informative meioses substantially increased our sample size, but as O’Connell *et al.* showed^12^, there was decreased power to call crossovers for these meioses, we adapted Model 2 to account for this by simply drawing the mother effects, *α*, from a distribution with a mean and variance that were specific to the family configuration *f* from cohort *c*:




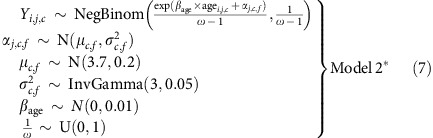




We fitted Model 2* to all meioses from families with at least two genotyped children; we excluded a particular family configuration for a cohort if it included fewer than 20 meioses. The family configurations used are explained in the section above on calling crossovers.

As for Model 1, we also fitted a more general version of Model 2 with cohort-specific age effects. The following model was fitted to the informative meioses:




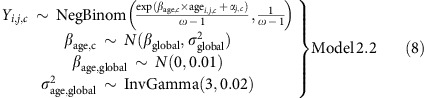




with the hyperpriors on *α*_*j*,*c*_ and the other priors the same as for Model 2. For the informative and partially informative meioses combined, we kept the *μ* and *σ*^2^ parameters cohort‐ and family configuration‐specific, but had a common *β*_age,*c*_ for all families in cohort *c*:




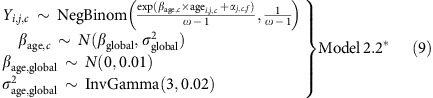




with the hyperpriors on *α*_*j*,*c*,*f*_ and the other priors the same as for Model 2*.

To check our results were robust to the choice of priors, we set each parameter in turn to have an uninformative prior, and fitted Model 2* again. Specifically, we used these priors:




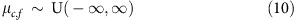







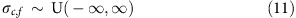







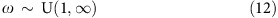







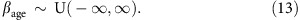




We chose a uniform prior on *σ*_*c*,*f*_ following the recommendation of Gelman^28^.

We fitted these models on the paternal crossovers too, using the same priors as above, with the following exceptions:




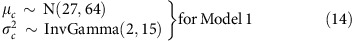







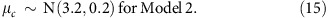




We fitted all models using a Markov Chain Monte Carlo (MCMC) method, specifically the No U-Turn Sampler extension to Hamiltonian Monte Carlo (HMC), implemented in RSTAN^29^. HMC takes a series of steps informed by the first-order gradient of the log-posterior to avoid the random walk behaviour inherent in other MCMC methods such as Metropolis and Gibbs sampling^30^.

We used four chains with 10,000 draws each, and discarded the first half as burn-in. Convergence was assessed using the 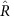 statistic, which measures the between- versus within-chain variance^31^. This should be close to 1 if convergence has been reached. If 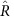 exceeded 1.05 for any parameter, the chains were run for a further 10,000 draws or until 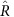 was less than 1.05. For some models, we also tried running the MCMC for 100,000 draws, discarded the first half, and thinned the remainder by taking every 100th draw. The 95% credible intervals were almost identical, and so we report results from 10,000 draws here.

### Posterior predictive checks

To assess the fit of these hierarchical models, we carried out posterior predictive checks to compare empirical properties of the observed data, *Y*, to data simulated under a model with our estimated parameters, which we denote *Y*^rep^. For all parameters in all models, the draws were concentrated in a single mode, and we took the draw with the highest posterior density as our Bayesian estimator. We denote the parameter estimates from this draw as 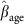, 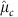 etc. Using these estimated parameters, we simulated 1,000 data sets of the same size as our actual data set, starting from the highest level of the hierarchy in the model. As an example, for Model 1, we simulated *α*_*j*,*c*_ for each mother from N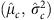, then simulated 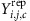 for each child from 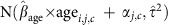. Then we compared these simulated data sets to the observed data by examining the empirical cumulative distribution functions and distributions of summary statistics that are ancillary to our inference. We also tried simulating data sets using 1,000 different sets of parameters drawn from the joint posterior distribution and comparing these to the observed data. Results were very similar to those from the simulations using the draw with the highest posterior density, so we show only results for the latter.

### Assessing the frequentist properties of the Bayesian method

We wanted to assess the probability that, if *β*_age_ had some true non-zero value *β*_age, true_, our Bayesian procedure would have estimated it well. We therefore estimated the coverage and power of the credible intervals under our Bayesian model. Formally, the 95% credible interval has a 100*γ*% coverage for a parameter *θ* if




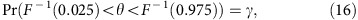




and 100*π* % power if




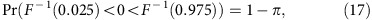




where *F*^−1^(*n*) is the 100*n*th quantile of the cumulative distribution function of the posterior of *θ*.

We selected 10 bins of *β*_age_ such that there were at least 1,000 draws in each bin, and then sampled, with replacement, 100 sets of parameters from among those draws. Note that we did this rather then specifying a particular value of *β*_age_ that we wanted to test, since we needed all the parameters in the model to be consistent. For each of those sets of parameters, we simulated a data set and fitted the model on it in RSTAN, using four chains with 10,000 draws. We then asked in what proportion of data sets the credible interval, at different levels, overlapped 0 (1−*π*) or overlapped the value of *β*_age_ used for simulation (*γ*).

## Additional information

**How to cite this article**: Martin, H. C. *et al.* Multicohort analysis of the maternal age effect on recombination. *Nat. Commun.* 6:7846 doi: 10.1038/ncomms8846 (2015).

## Supplementary Material

Supplementary Information Supplementary Figures 1-17, Supplementary Tables 1-7 and Supplementary Note 1

## Figures and Tables

**Table 1:** Cohorts included in the study.

**Cohort**	**Number of samples**	**Duos from informative nuclear families**	**Informative duos**	**Duos from families with** >1 **child**
Carlantino, Italy	630	6 (2)	29 (11)	53 (23)
French Canadians	477	106 (29)	158 (50)	218 (80)
Friuli Venezia Giulia, Italy	1,236	13 (4)	72 (26)	160 (70)
Korcula, Croatia	897	0 (0)	7 (3)	34 (17)
Netherlands Twin Registry	2,729	298 (96)	398 (130)	889 (379)
ORCADES, Orkney, Scotland	2,215	48 (15)	118 (40)	252 (109)
Queensland Twin Registry (QTR370)	3,754	890 (239)	1,310 (323)	1,441 (389)
Queensland Twin Registry (QTR610)	7,364	1,283 (403)	1,337 (420)	2,898 (1202)
Queensland Twin Registry (QTRCoreExome)	4,444	234 (75)	329 (106)	781 (332)
Val Borbera, Italy	1,664	30 (9)	72 (26)	266 (123)
Vis, Croatia	960	0 (0)	11 (4)	37 (17)
Total		3,036 (910)	4,253 (1268)	7,688 (3003)
Total analysed		2,889 (866)	3,823 (1132)	6,011 (2305)

The three rightmost columns show the number of maternal meioses for which we had age data, followed by the number of families in parentheses. The total number analysed excludes the cohorts (or, for the rightmost column, family configurations within a cohort) with fewer than 20 meioses. The SNP chips used, number of SNPs and sample sizes for paternal meioses are given in Supplementary Table 1.

**Table 2:** Summary of posteriors for *β*_age_ for different Bayesian models.

**Parent**	**Model**	**2.5%**	**25%**	**50%**	**75%**	**97.5%**	**Pr(***β*_age_ >0)
Maternal	Model 1	0.02672	0.06608	0.08582	0.10706	0.14513	0.99803
Maternal	Model 2	1.00081	1.00171	1.00217	1.00266	1.00367	0.99963
Maternal	Model 2*	1.00098	1.00172	1.00213	1.00251	1.00333	0.99995
Paternal	Model 1	−0.04766	−0.02582	−0.01492	−0.00410	0.01769	0.17883
Paternal	Model 2	0.99820	0.99906	0.99951	0.99997	1.00089	0.23465
Paternal	Model 2*	0.99849	0.99925	0.99965	1.00006	1.00082	0.28315

This table shows several quantiles of the posterior of *β*_age_ for Model 1 and of 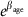 for Models 2 and 2*, as well as the posterior probability that *β*_age_ is greater than 0. Models 1 and 2 were fitted to fully informative meioses, and Model 2* to fully and partially informative meioses. Note that the interpretation of *β*_age_ is additive for Model 1 and multiplicative for Models 2 and 2*. These results are for duoHMM counts.
