# Supplementary material for: Multicohort analysis of the maternal age effect on recombination
Source: Nat Commun. 2015 Aug 5;6:7846. doi: 10.1038/ncomms8846 (PMC4580993; doi:10.1038/ncomms8846)
Supplement: Supplementary Information — Supplementary Figures 1-17, Supplementary Tables 1-7 and Supplementary Note 1 [file ncomms8846-s1.pdf]

Supplementary Figures

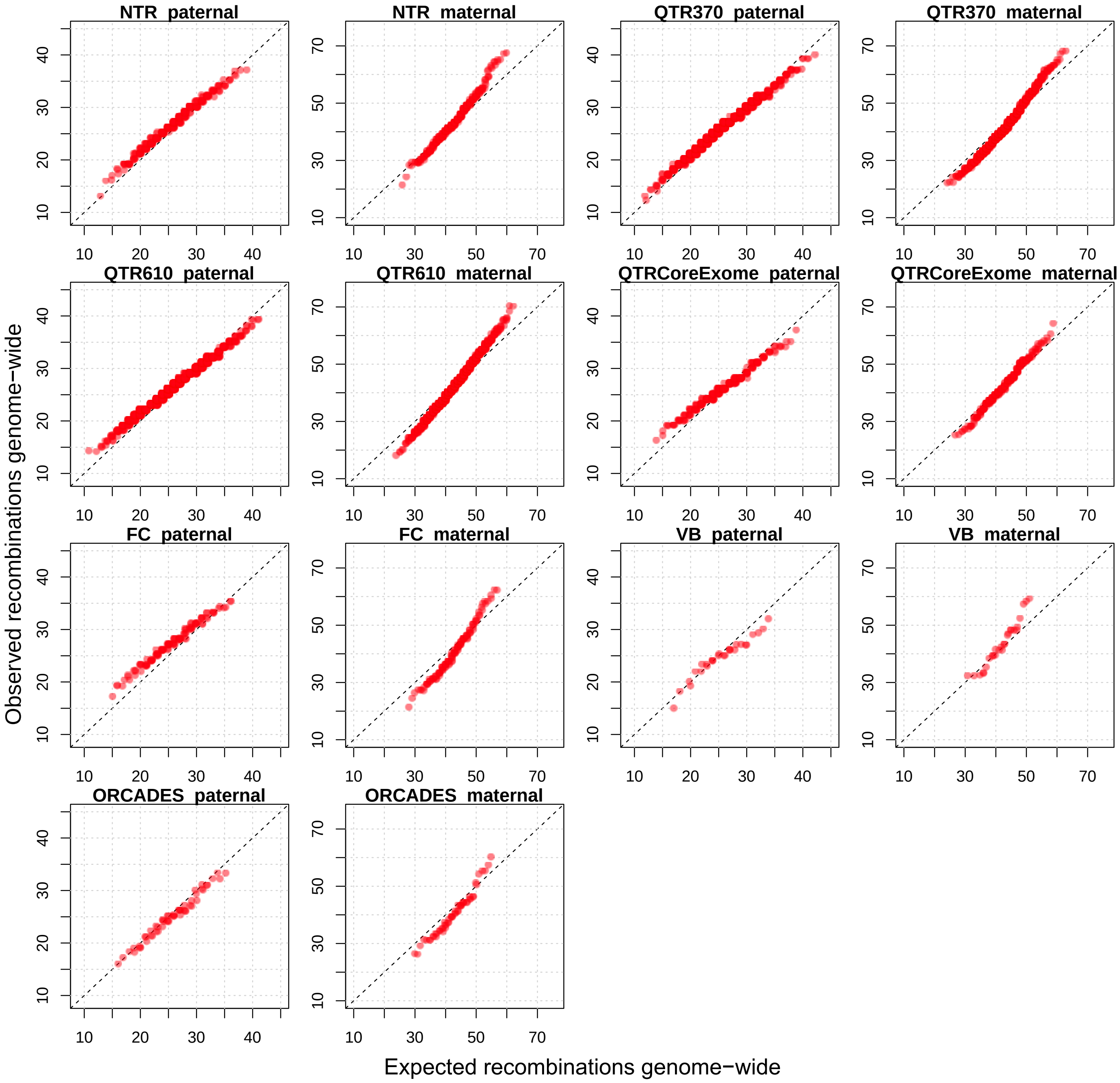

Supplementary Figure 1. Distribution of number of crossovers called by NFTOOLS. QQ-plots for the observed versus expected number of crossovers called by NFTOOLS for paternal and maternal meioses in each cohort. The expected counts were generated from a Poisson distribution using the genetic lengths from the deCODE 2002 map [32]. Note that the points have been jittered to aid visualisation, so the counts do not always appear to be strictly increasing.

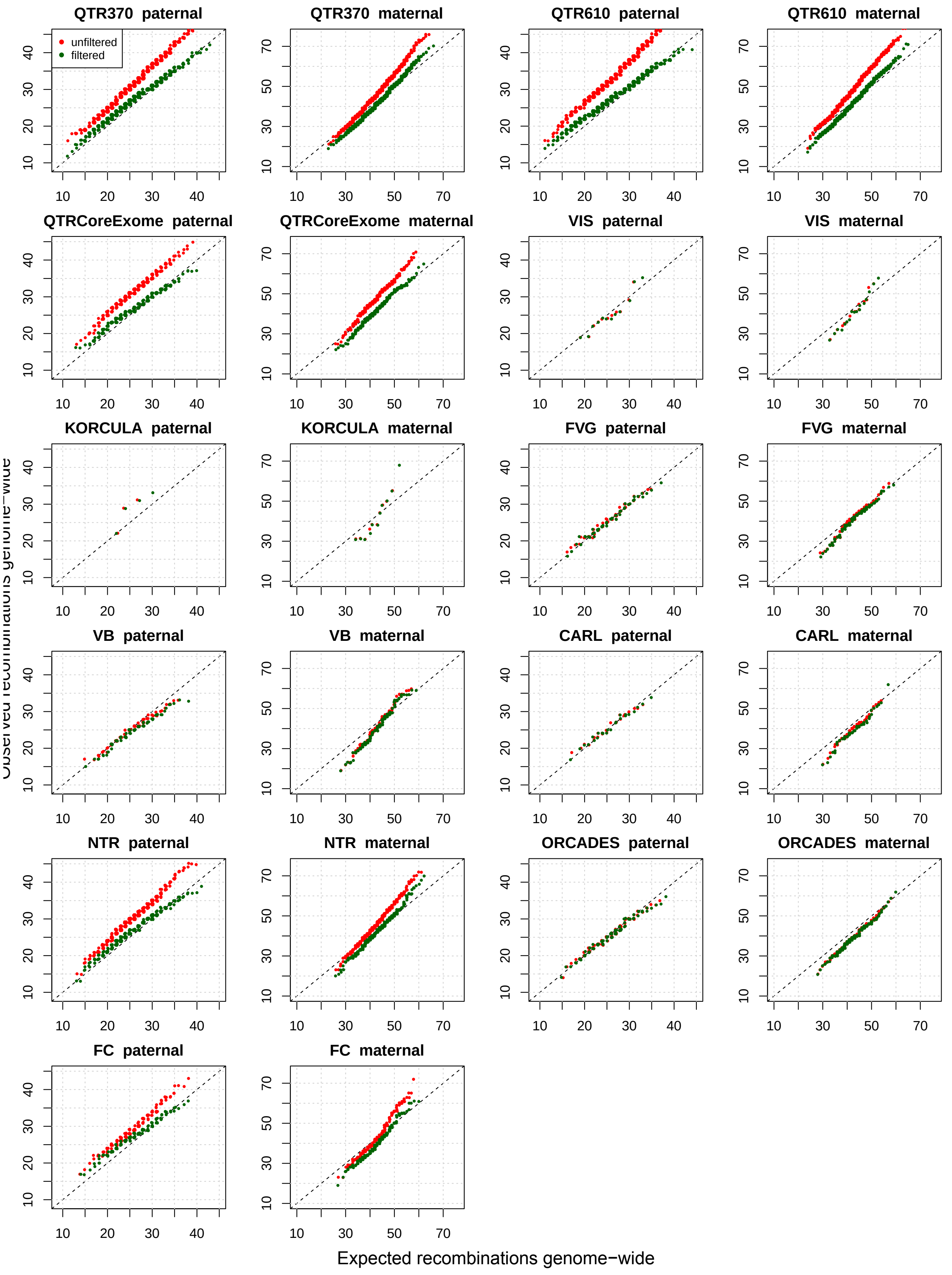

Supplementary Figure 2. Distribution of number of crossovers called by duoHMM, before and after filtering. The qq-plots were constructed as for Supplementary Figure 1, but using data from informative meioses called with duoHMM.

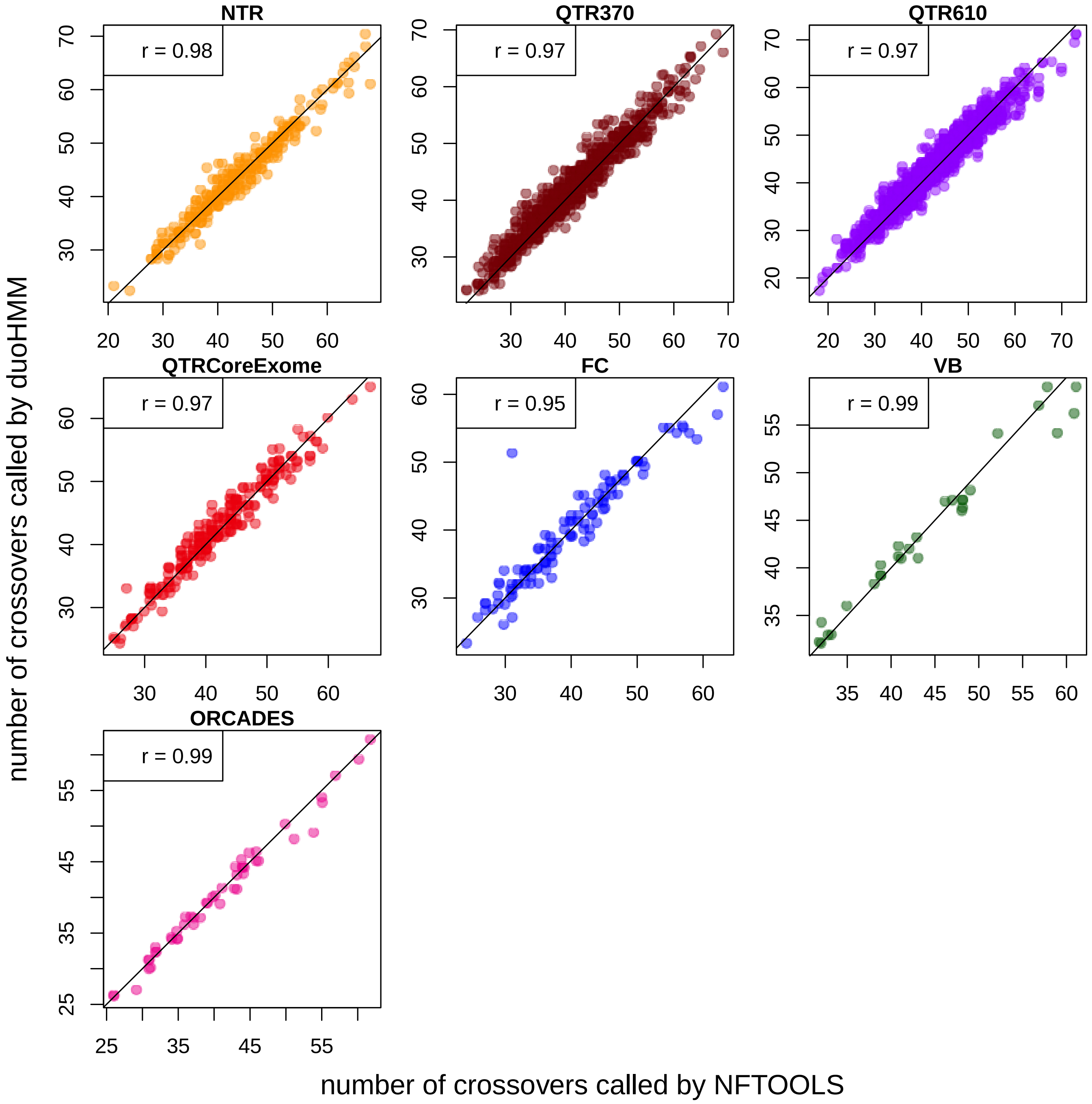

Supplementary Figure 3. Comparison of number of maternal crossovers called by duoHMM versus NFTOOLS in informative nuclear families. The Pearson correlation is shown in the top left.

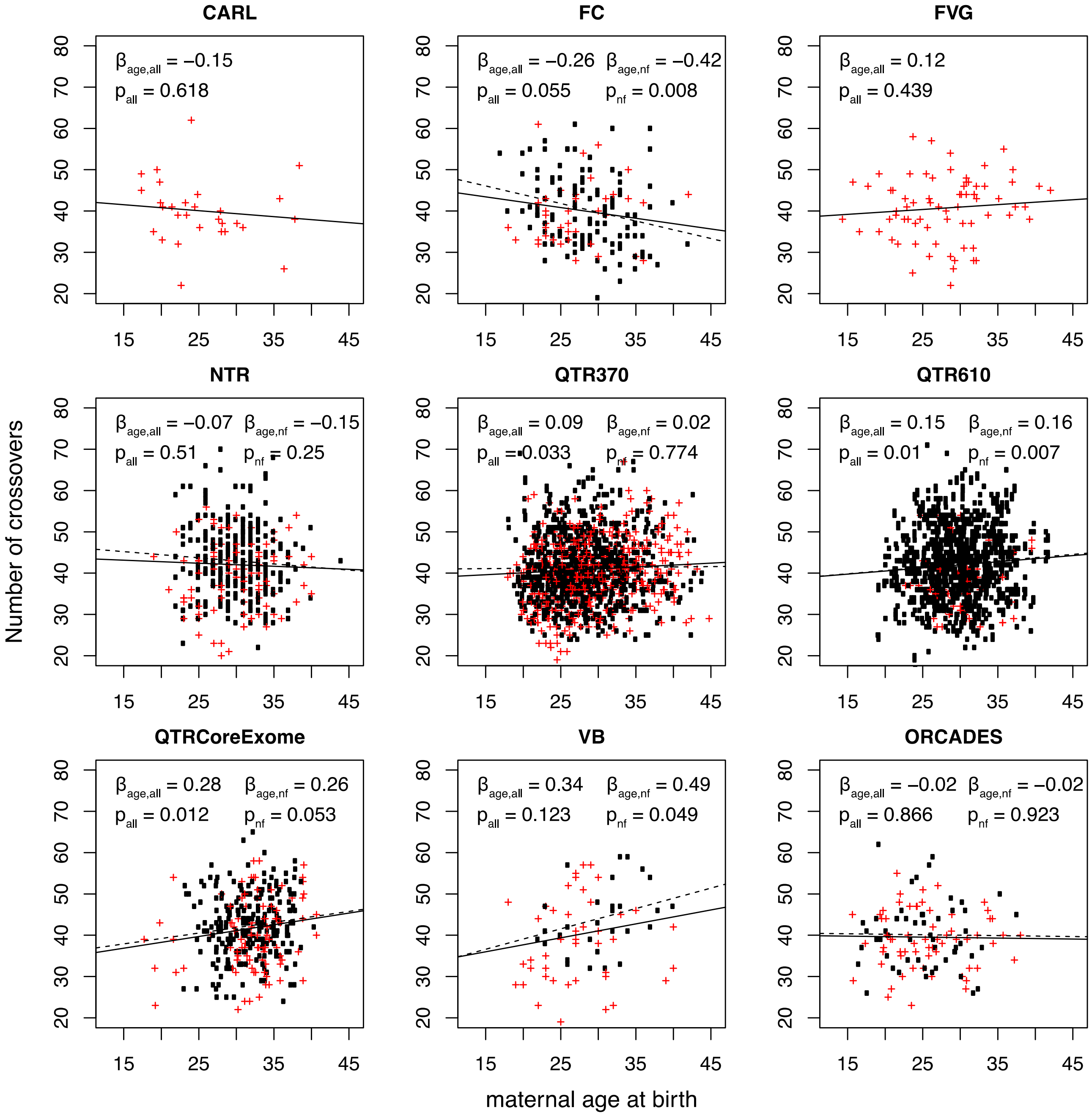

Supplementary Figure 4. Scatter plots of the number of crossovers versus maternal age for each cohort. Counts are from duoHMM. Meioses from informative nuclear families (as could be analysed by NFTOOLS) are shows as black dots, and those from families with only two children but a third generation (fully informative for duoHMM) as red crosses. The lines are from a linear mixed model on the number of crossovers versus maternal age, fitted to either all fully informative meioses (solid line) or only to meioses from informative nuclear families (dotted line). The corresponding slopes and their p-values (from a t-test in the linear mixed model) are indicated on the left for all fully informative meioses (p_{all}) and on the right for meioses from informative nuclear families (p_{nf}).

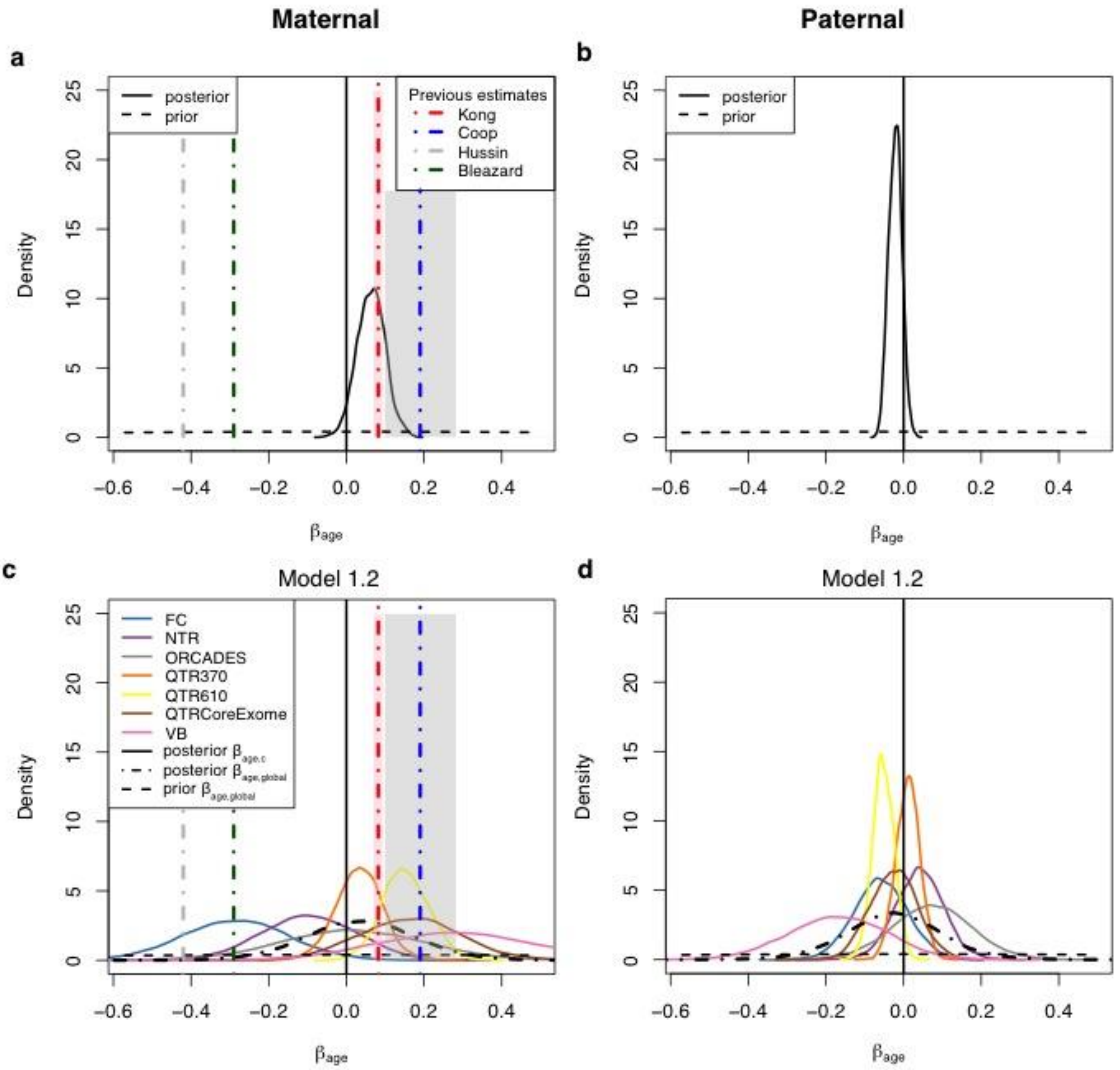

Supplementary Figure 5. Bayesian posteriors for the age effect, with NFTOOLS data. These plots show the priors and posteriors for β_{age} from Models 1 and 1.2 fitted to the number of crossovers called in informative nuclear families by NFTOOLS. For Model 1.2, the posterior for $\beta_{age,global}$ is also indicated. The vertical lines show the estimates from previous studies, and the shaded boxes the corresponding standard errors, if reported. Note that these results are based on meioses from informative nuclear families, where as Figure 3 is based on all informative meioses.

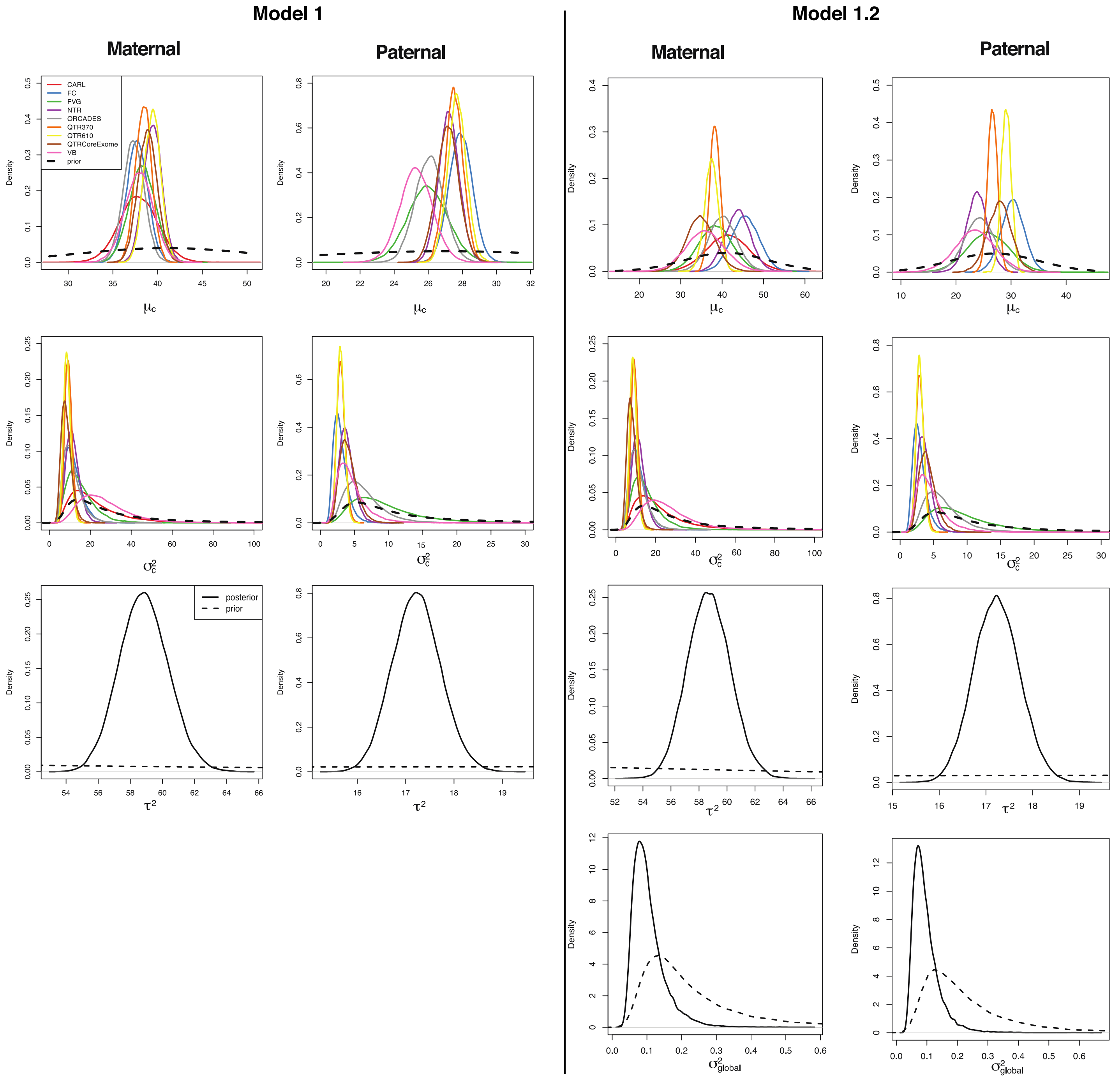

Supplementary Figure 6. Bayesian posteriors for parameters from Models 1 and 1.2, for duoHMM data. We fitted a normal model on the number of crossovers called in fully informative meioses, with either the same or different age effects for all cohorts (Model 1 or 1.2; see Methods). The posteriors for the age effects are shown in Figure 3. In this figure, we show the posteriors for μ_c and $\sigma^2{}_c$, the cohort-specific mean and variance of the parent effects respectively, τ^2, the residual variance, and $\sigma^2{}_{age,global}$, the variance of the cohort-specific age effects.

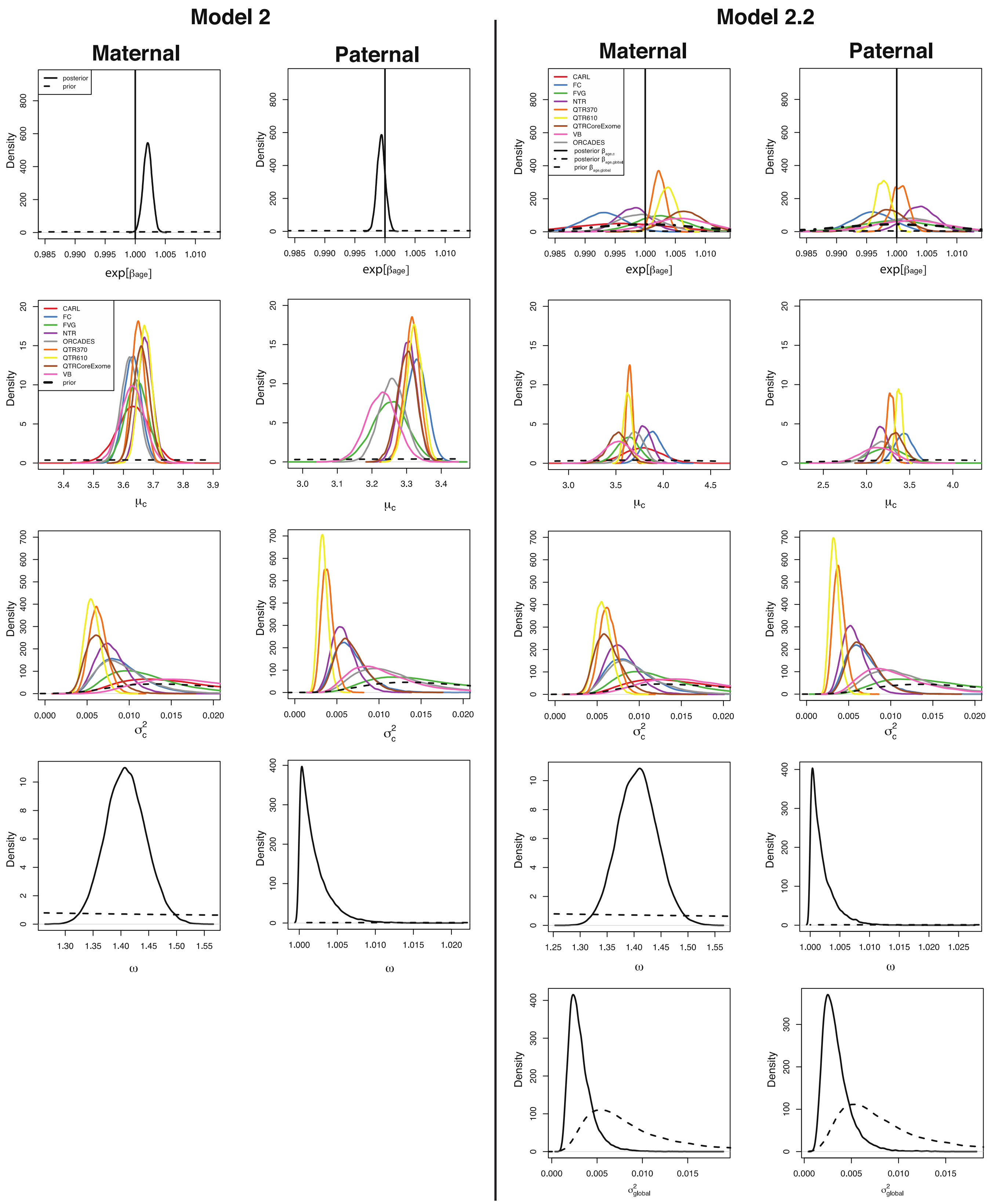

Supplementary Figure 7. Bayesian posteriors from a negative binomial model fitted to fully informative meiosis. These plots show results from Models 2 and 2.2, described in the Methods, fitted to duoHMM counts from informative duos. Parameters are as described for Figure 4 and Supplementary Figure 6, except that ω is the over-dispersion parameter, replacing τ^2 from the normal model. Note that the expected number of crossovers increases by $\exp[\beta_{age}]$-fold per year.

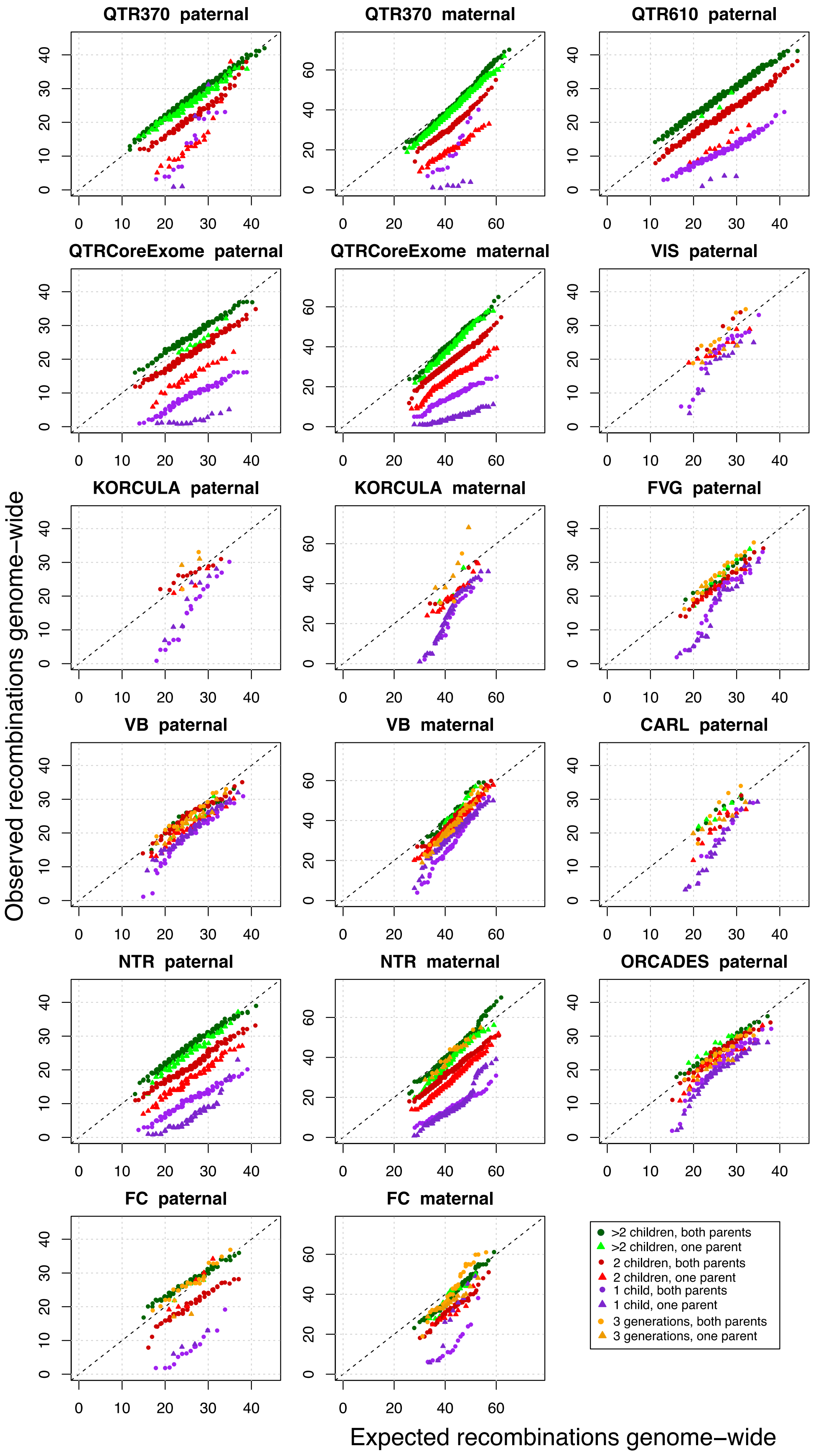

Supplementary Figure 8. Crossover distributions by cohort, called by duoHMM, for different configurations of family members genotyped. The qq-plots were constructed as for Supplementary Figure 1, but using filtered duoHMM calls. The number of crossovers called is in line with expectation for fully informative meioses, but somewhat lower than expected for families in which fewer than three children were genotyped. The extent to which this happens depends on the cohort, reflecting differences in sample size and in the amount of low-level relatedness, since these factors affect the phasing accuracy.

Supplementary Figure 9A

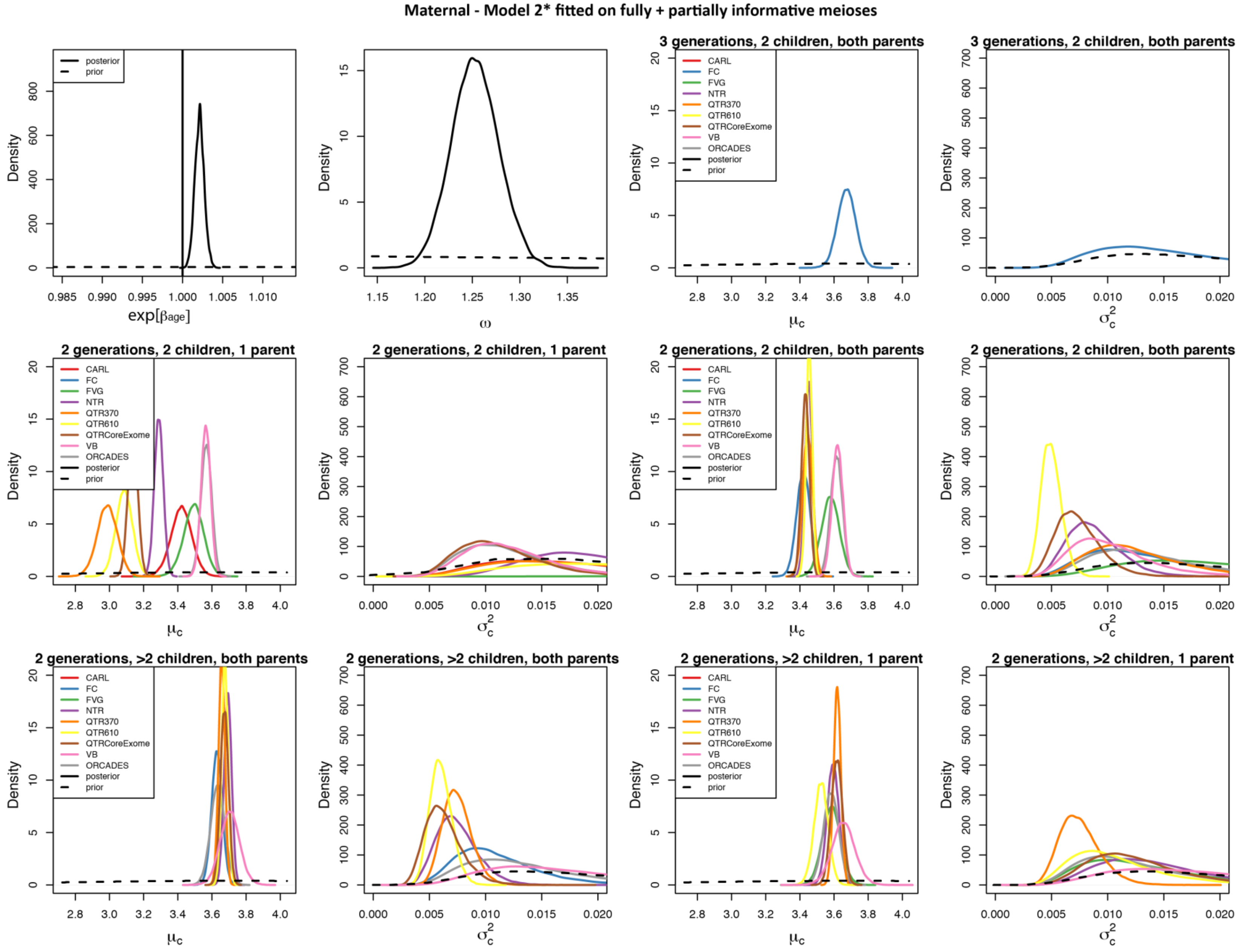

Supplementary Figure 9B

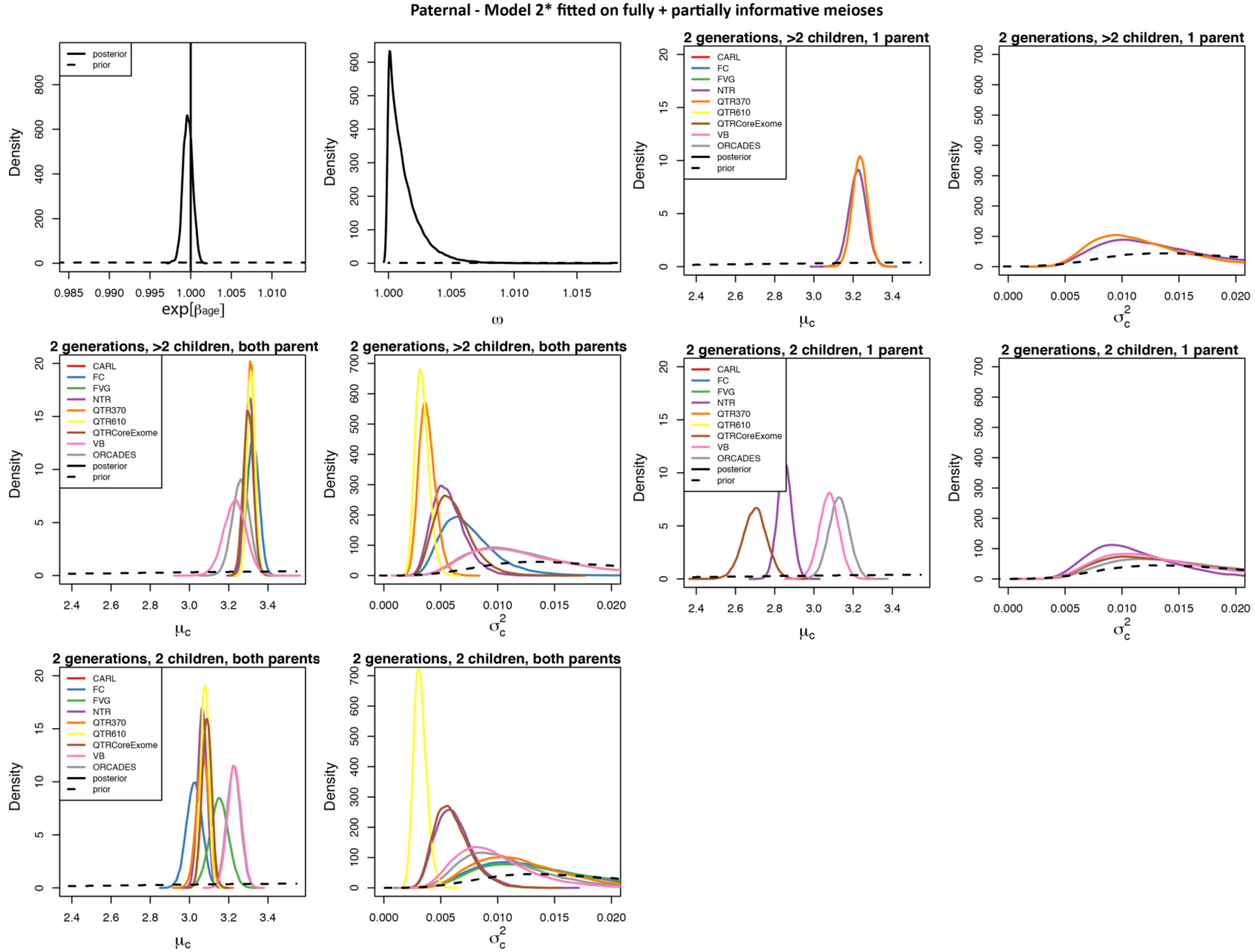

Supplementary Figure 9. Bayesian posteriors from a negative binomial model fitted to fully and partially informative meioses. This is from Model 2* (see Methods) fitted to maternal (A) or paternal (B) crossovers from all families with more than one child. Parameters are as described for Model 2 in Supplementary Figure 7, except that there are different μ and σ for each family type-cohort combination. As for Model 2, note that the expected number of crossovers increases by exp[β_{age}]-fold per year.

Supplementary Figure 10A

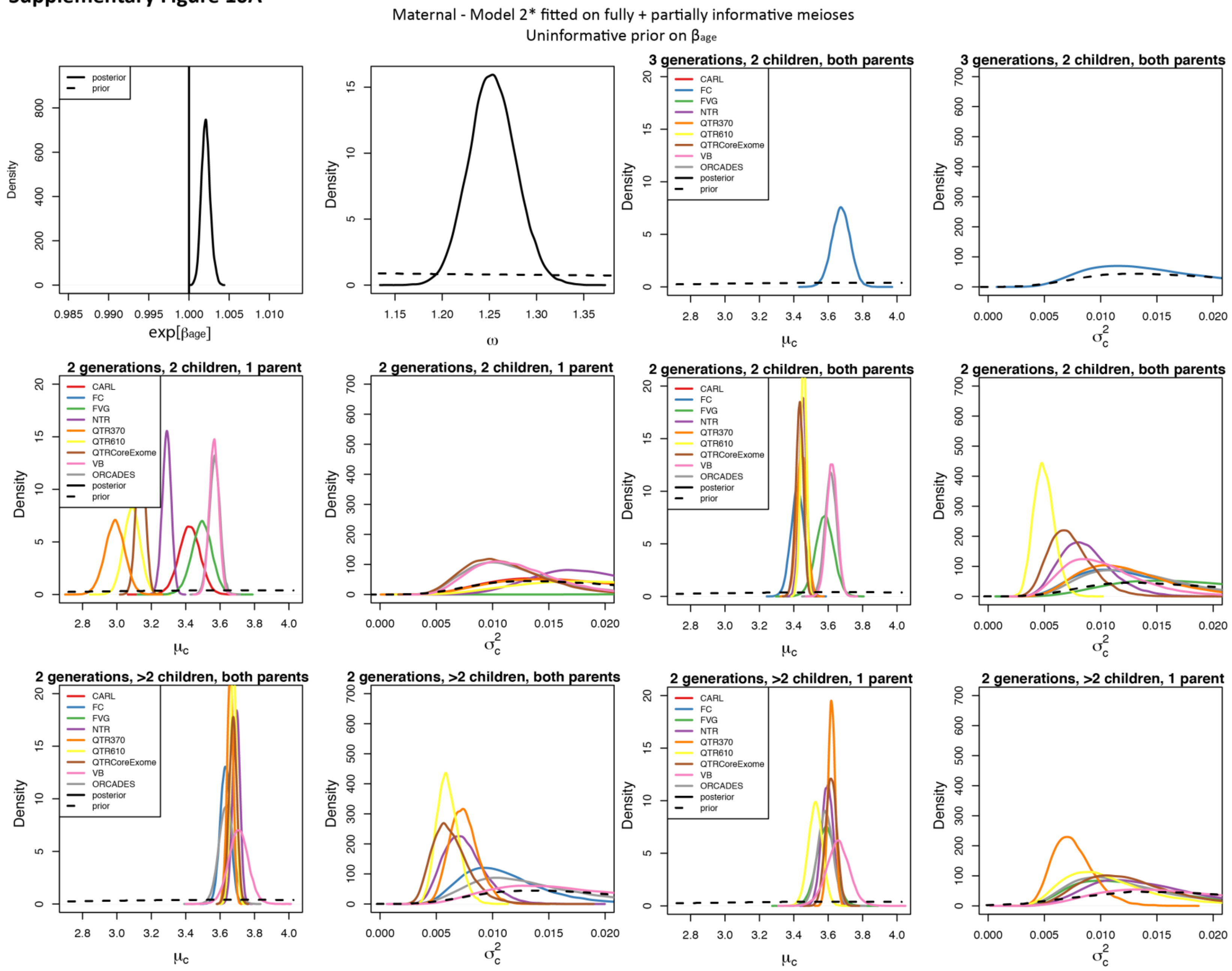

Supplementary Figure 10B

Maternal - Model 2* fitted on fully + partially informative meioses
Uninformative prior on μ_c

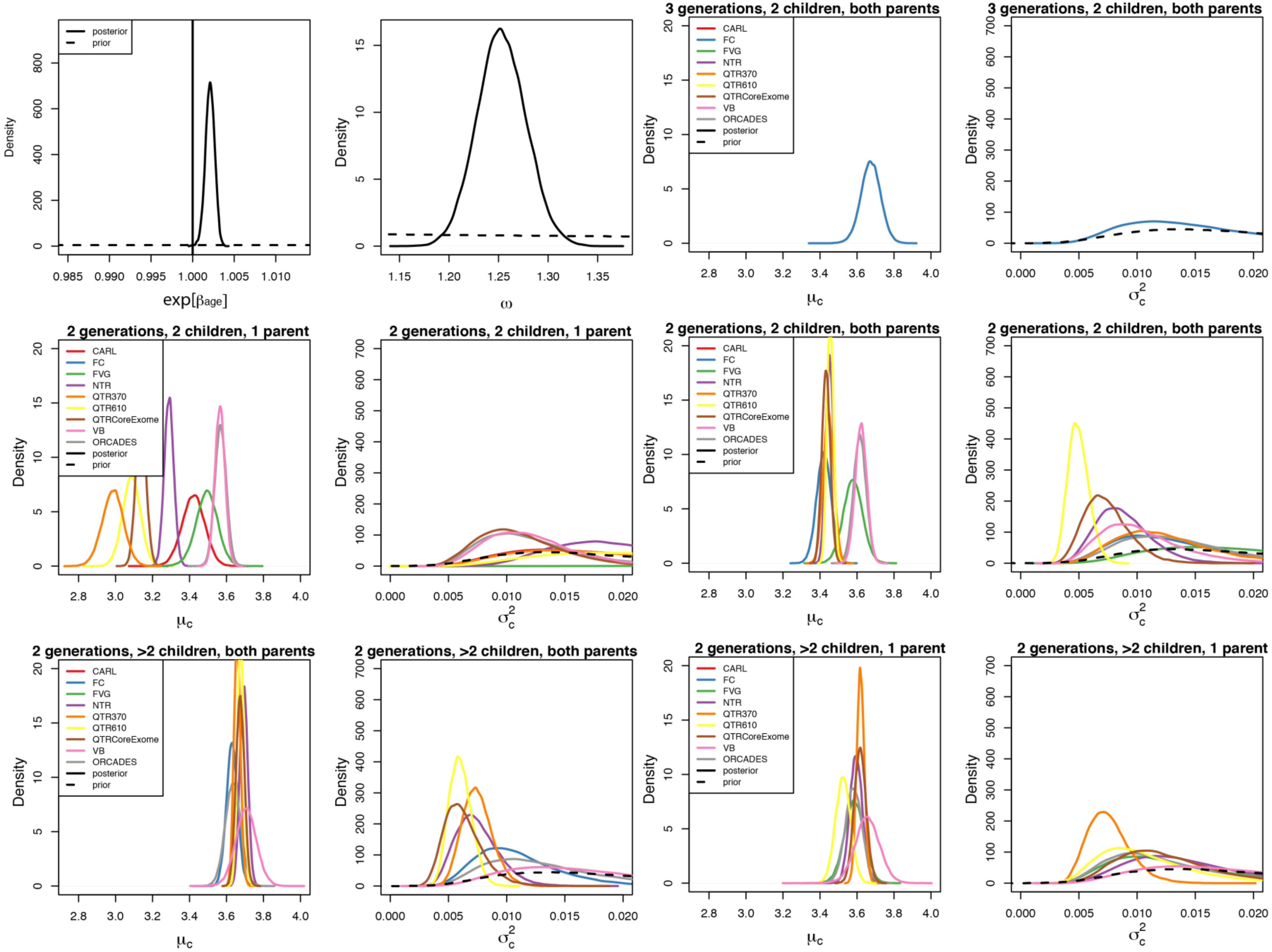

Supplementary Figure 10C

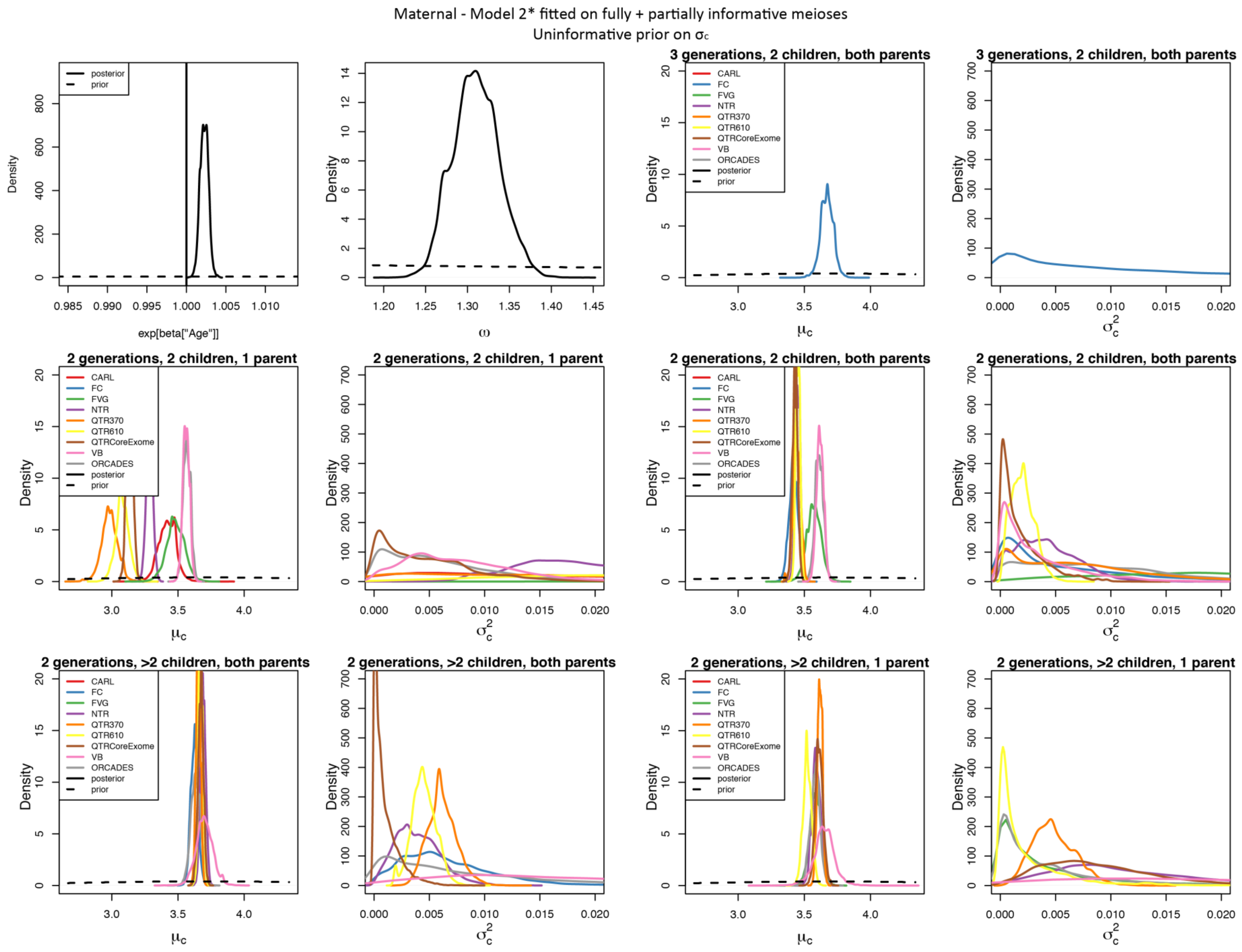

Supplementary Figure 10D

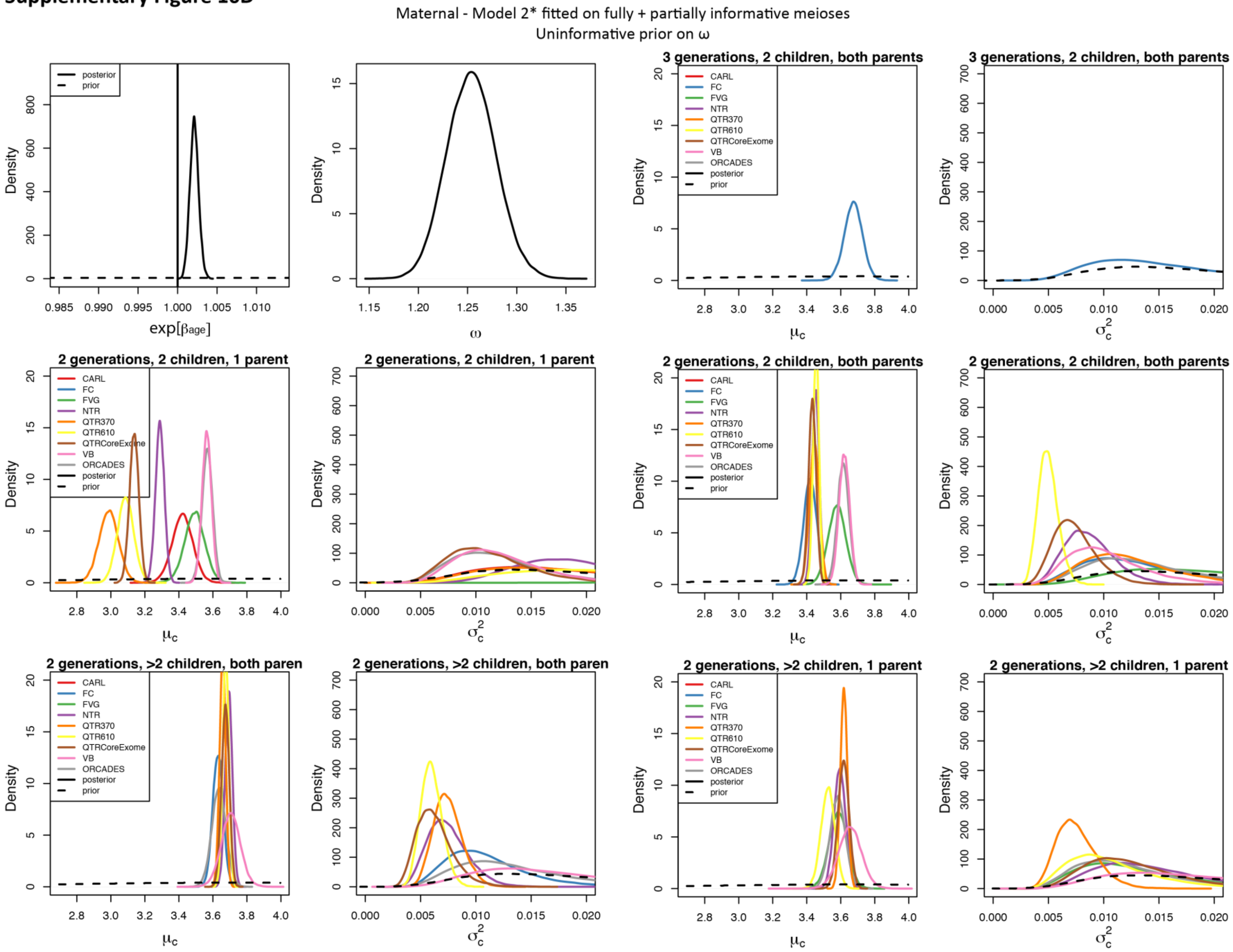

Supplementary Figure 10. Bayesian posteriors from a negative binomial model fitted to fully and partially informative meioses (Model 2*), with uninformative priors. As for Supplementary Figure 9, except that we set uninformative priors on β_{age} (A), μ_c (B), σ_c (C) and ω (D) in turn.

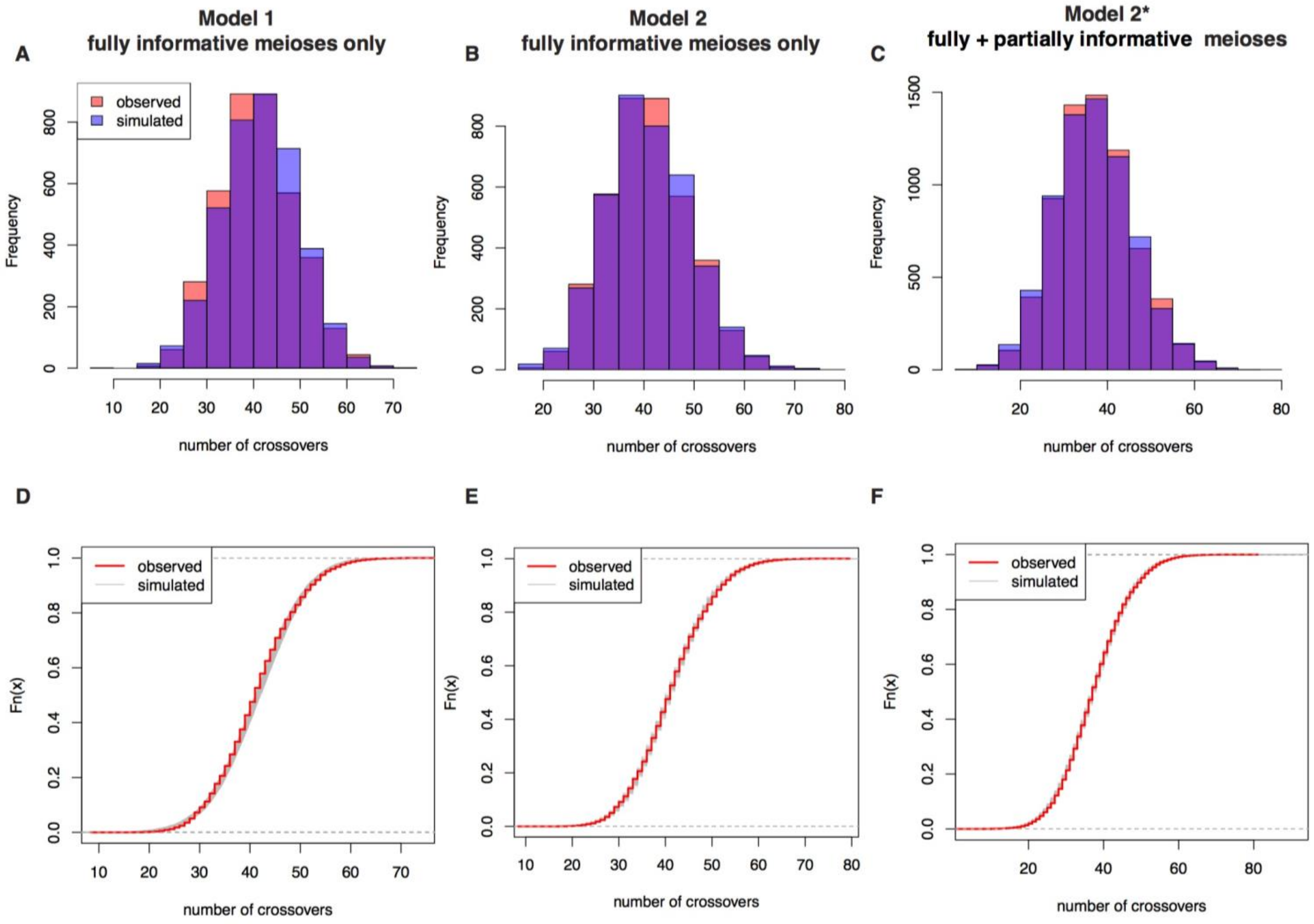

Supplementary Figure 11. Replications of the maternal crossover counts from the posterior predictive distributions, compared to observed data. We took the parameter estimates from the draw with the highest log probability, and simulated, from the highest level of the hierarchy, 1000 datasets of the same size as our actual dataset under the model (duoHMM counts for fully informative meioses). Panels A-C show histograms of the observed data and a randomly selected simulated dataset. Panels D-F show empirical cumulative distribution functions for 100 randomly selected datasets, compared to the observed data.

Supplementary Figure 12

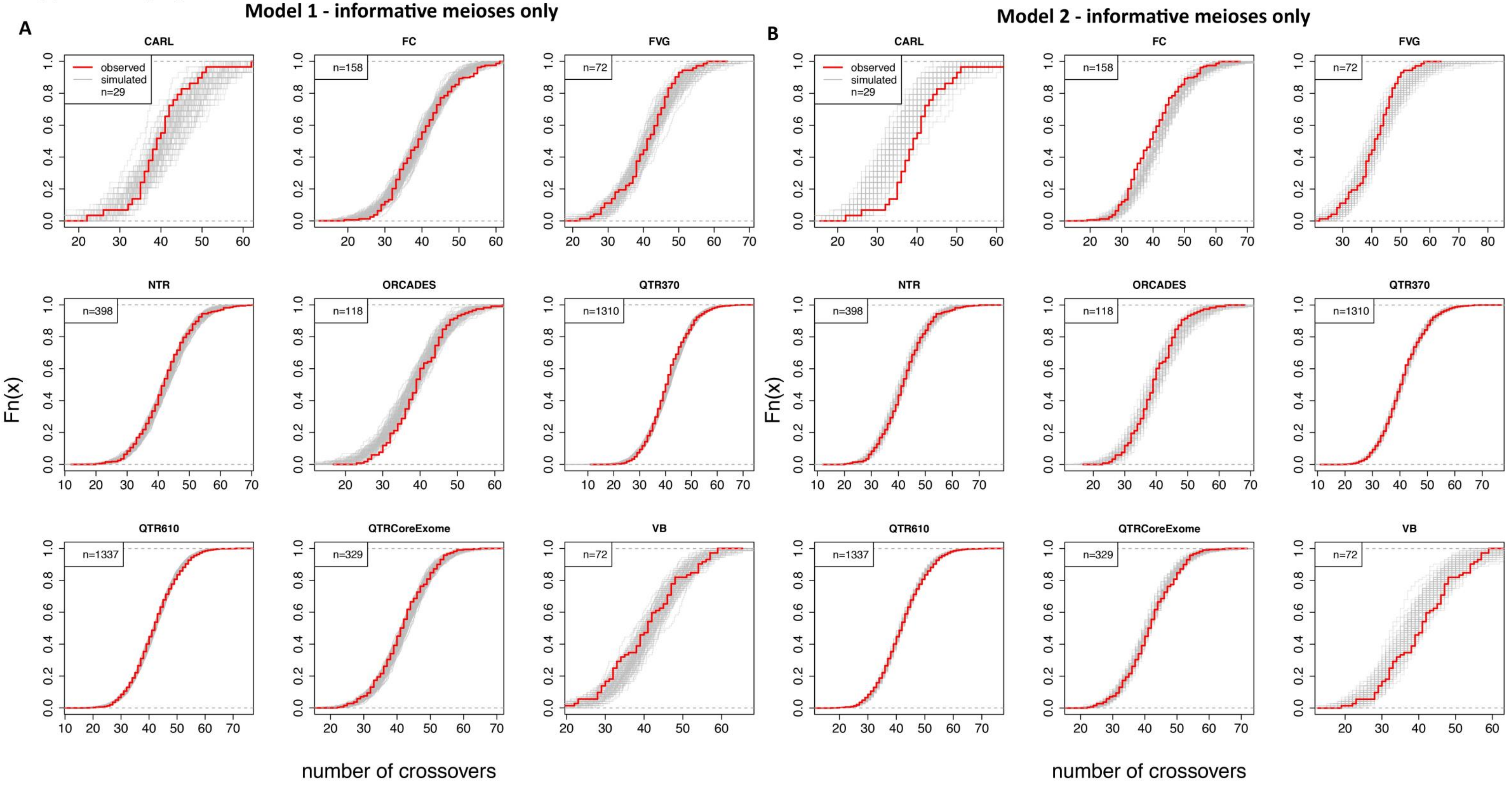

Supplementary Figure 12
C

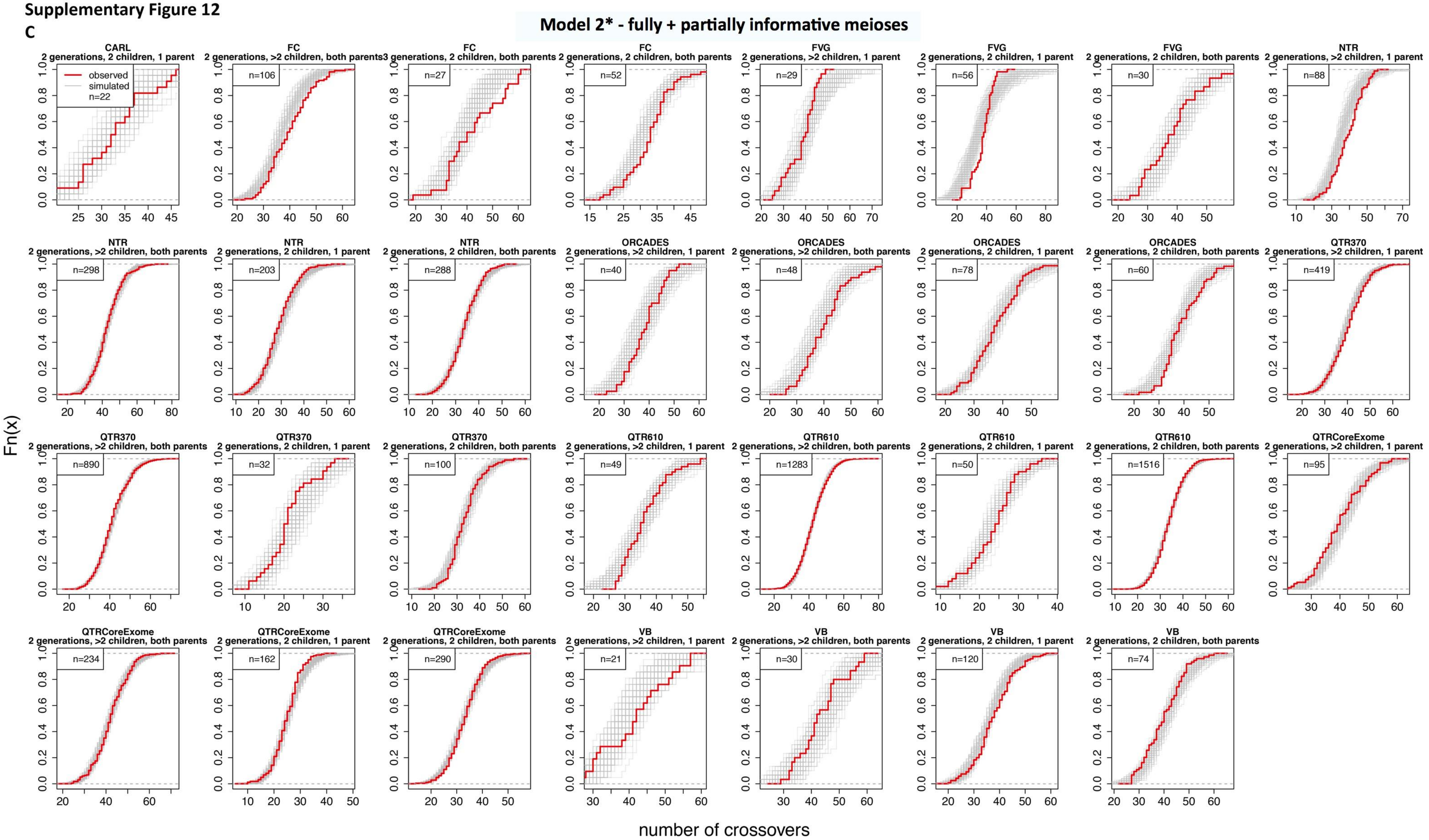

Supplementary Figure 12. Empirical cumulative distribution functions for 100 posterior predictive simulated datasets, by cohort. As for Supplementary Figure 11D-F, but with the samples stratified by cohort.

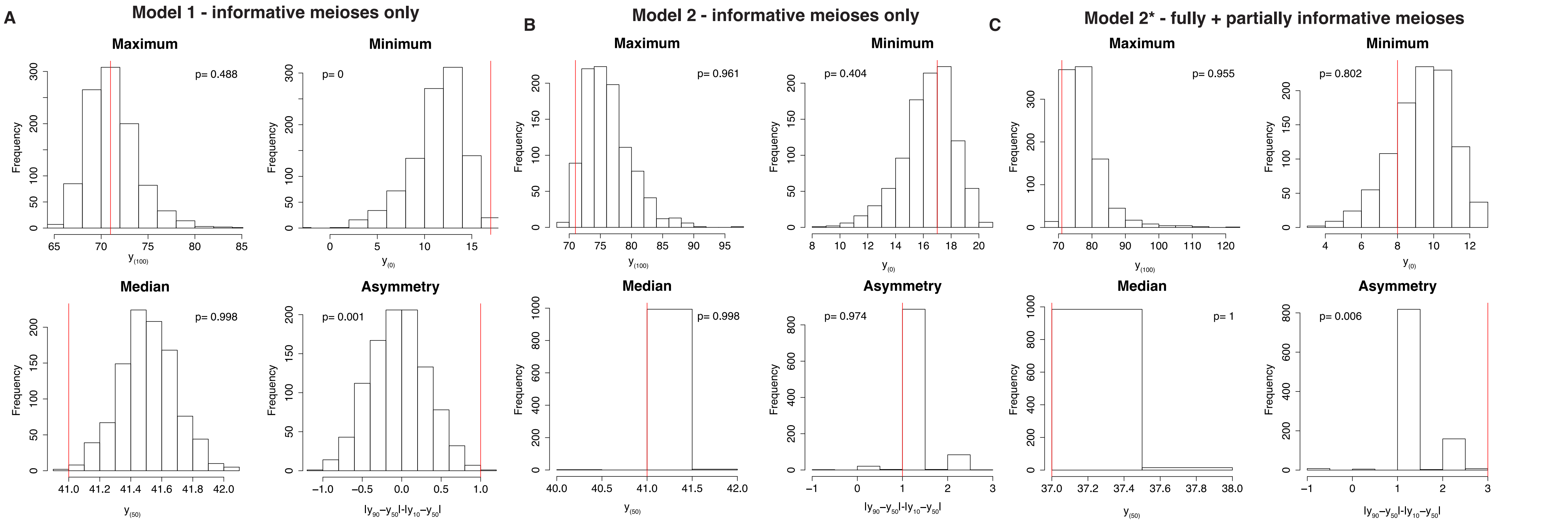

Supplementary Figure 13. Distributions of summary statistics for datasets simulated from posterior predictive distributions. Histograms of an asymmetry statistic and the maximum, medium and median crossover counts for the simulated datasets shown in Supplementary Figure 11D-F. The asymmetry is defined as $|y_{90}-y_{50}|-|y_{10} - y_{50}|$, where y_i is the *i*th percentile of the distribution of the number of crossovers. Thus, it should be 0 for a symmetric distribution. The red line indicates the value from the observed data, and *p* is an empirical p-value, specifically, the proportion of simulated datasets with a statistic great than or equal to the observed value.

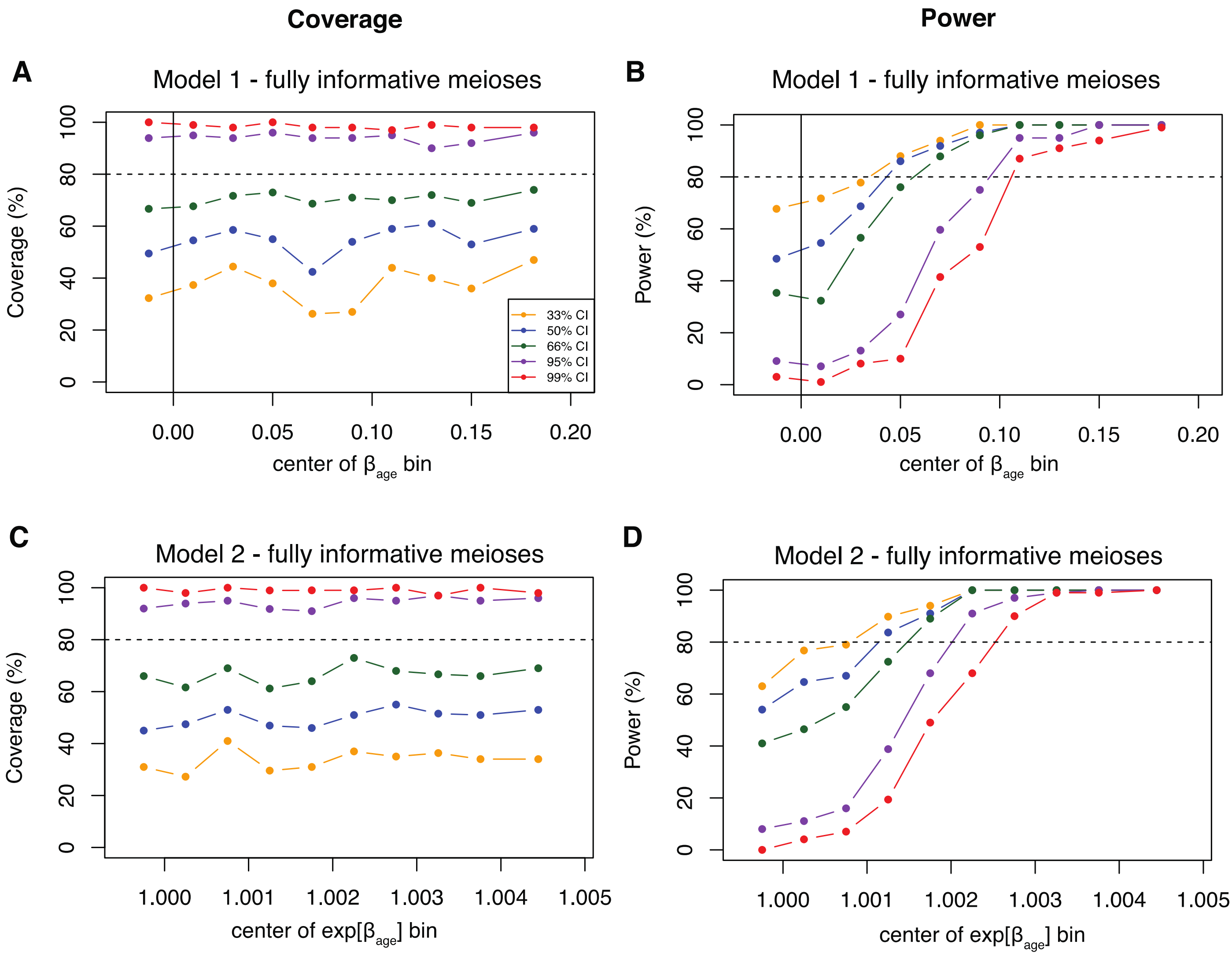

Supplementary Figure 14. Frequentist properties of our Bayesian procedure. We divided β_{age} into 10 bins based on the posterior, sampled 100 draws from each bin, and used those parameters to simulate datasets on which we then refitted the Bayesian model. The left-hand plots show the coverage, or percentage of simulations in which the indicated credible interval overlapped the value of β_{age} used for simulation. The coverage for the bin centred on 0 is equivalent to the Type I error. The right-hand plots show the power, or percentage of simulations in which the interval did not overlap 0.

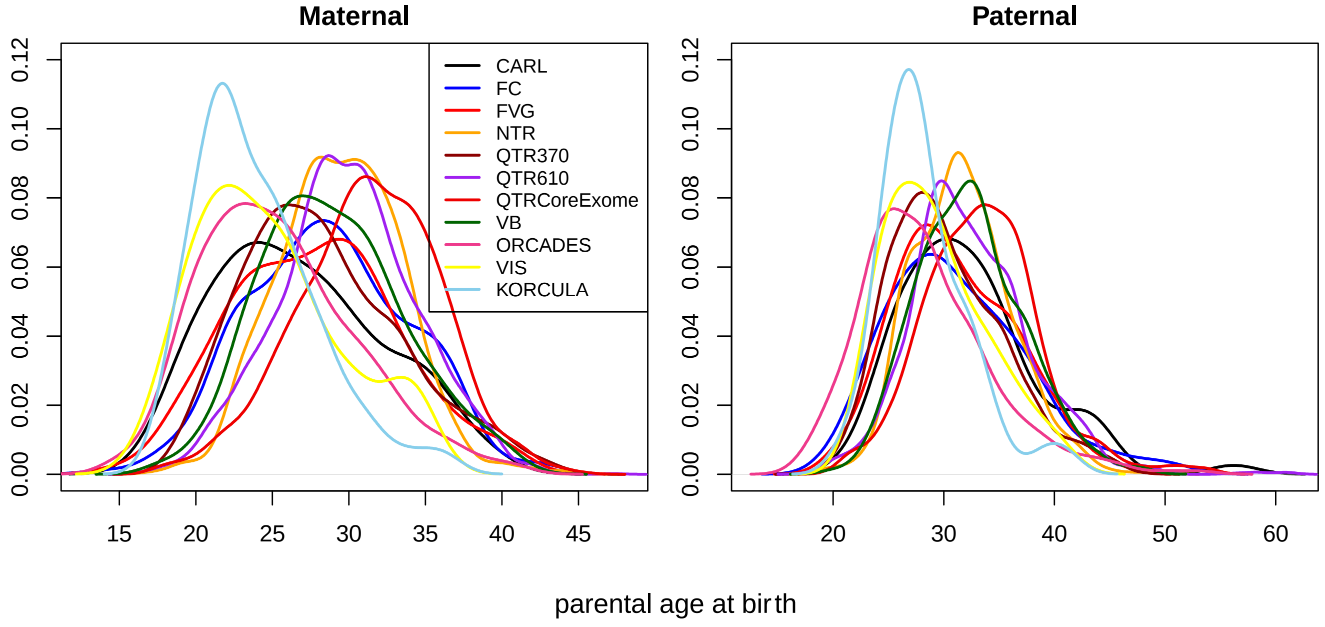

Supplementary Figure 15. Distributions of parental age at birth by cohort.

Supplementary Figure 16

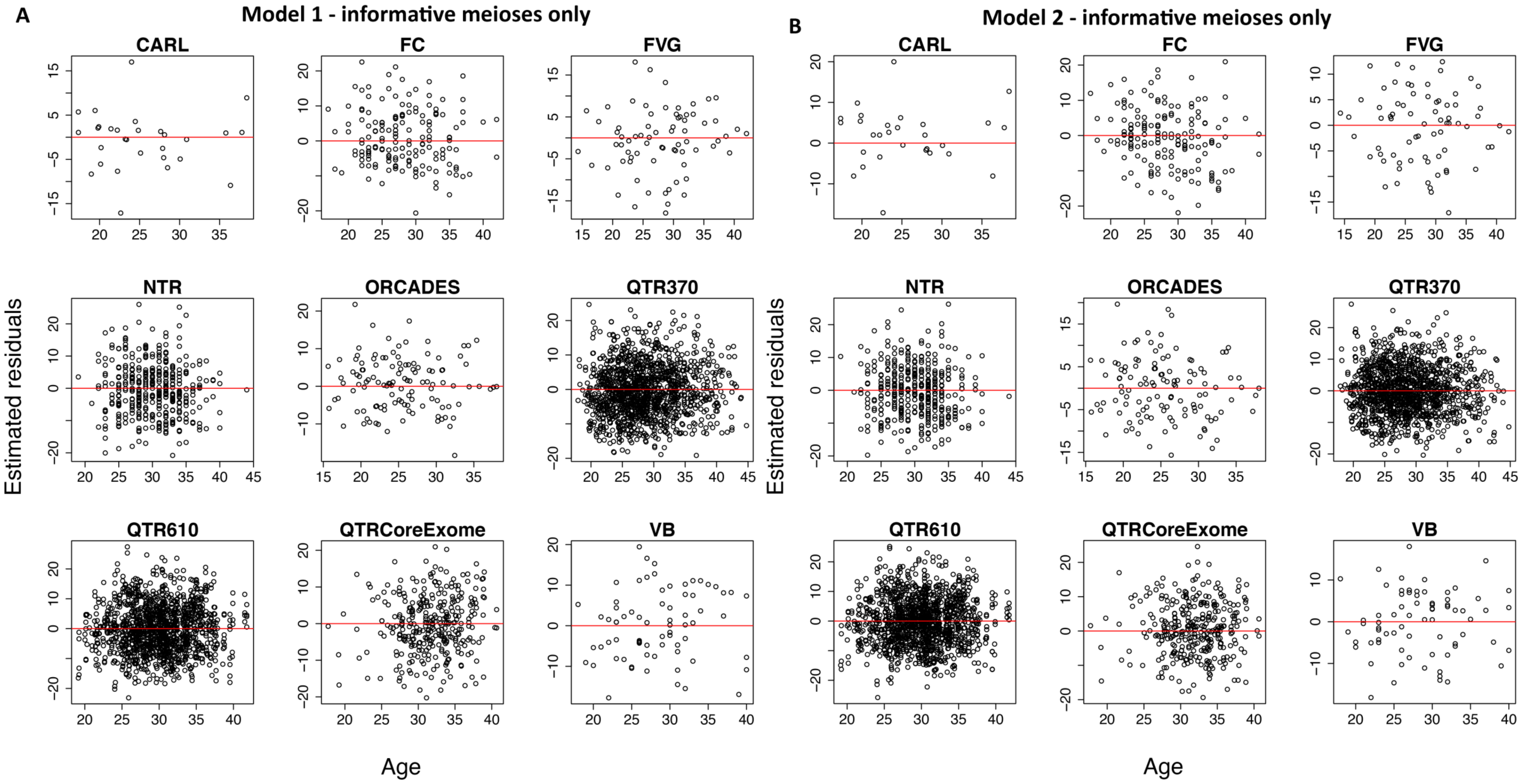

Supplementary Figure 16
C

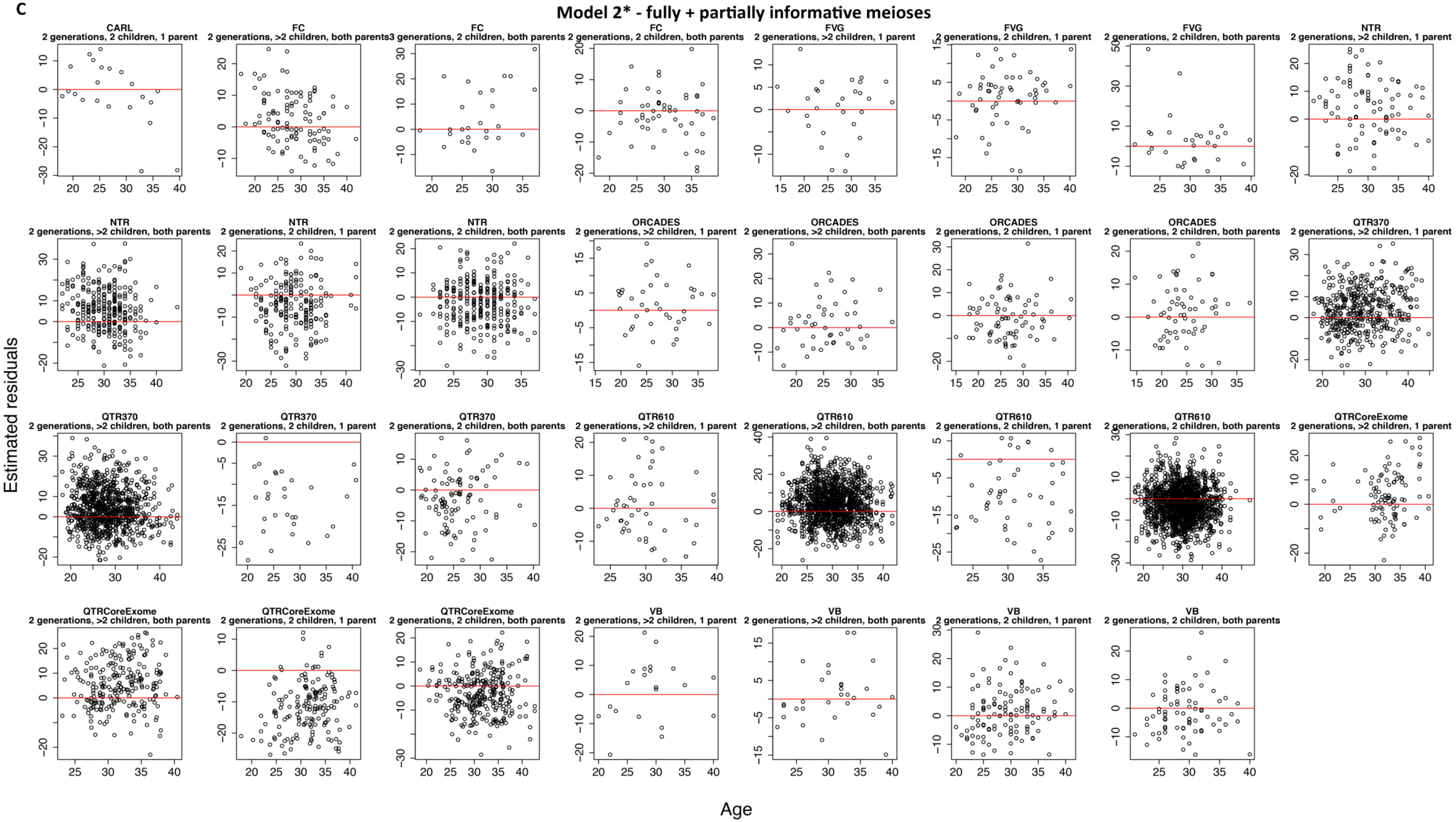

Figure 16. Plots of residuals *versus* maternal age. The residuals were calculated by subtracting the number of crossovers expected (given the parameters for the draw with the highest likelihood) from the observed number of crossovers.

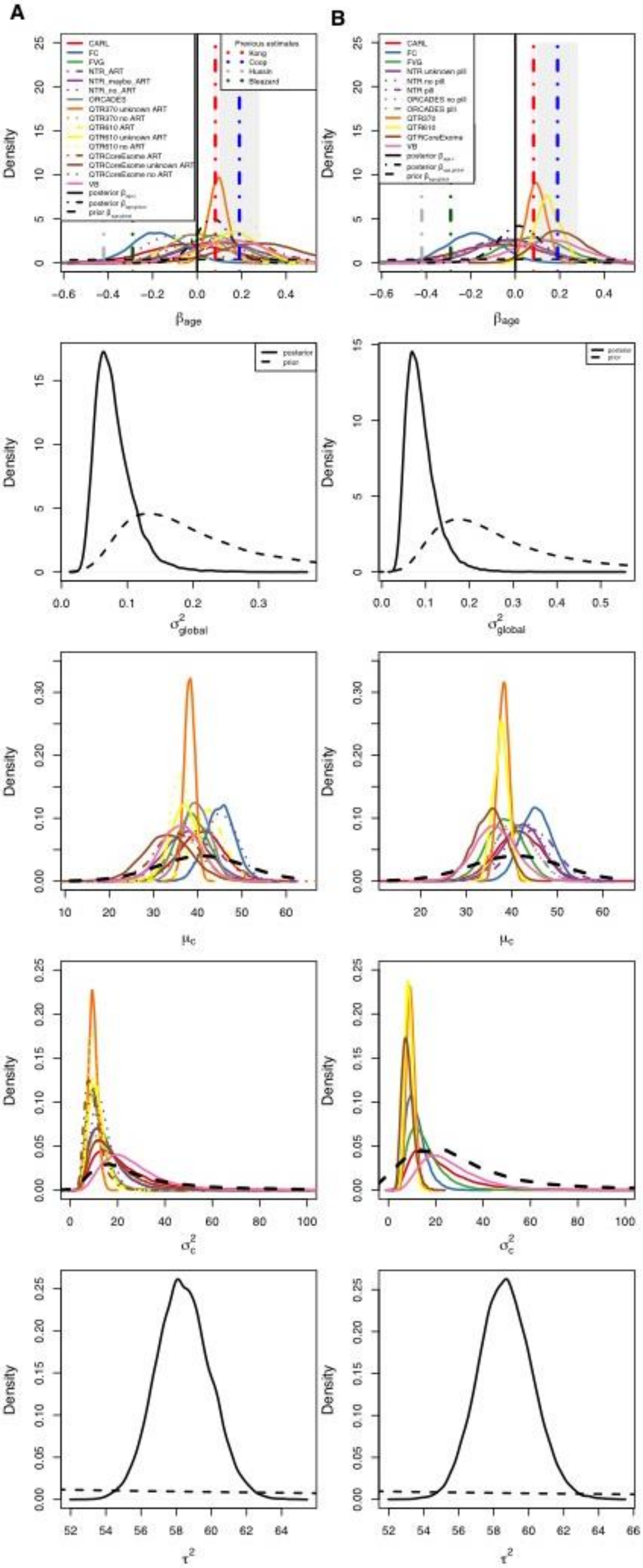

Supplementary Figure 17. Bayesian posteriors for parameters from Model 1.2, with mothers stratified by usage of alternative reproductive technologies (ART) or by oral contraceptive (OC) usage. A) QTR and NTR mothers are stratified into those who used ART, those who did not, and those for whom we did not have the information, or who had given conflicting answers at different times. B) NTR and ORCADES mothers are stratified into those who had taken oral contraception before the birth of their children (OC), those who had not (no OC) and those for whom this information was not available (unknown OC). Parameters are as defined in Figure 3 and Supplementary Figures 6 and 7. We fitted the model to the duoHMM data.

Supplementary Tables

Supplementary Table 1. Number of paternal meioses, by cohort. These are slightly lower than the number of maternal meioses given in Table 1, because age data were not always available for the fathers for whom we had genotype data. Also shown are the genotyping chip and number of SNPs used for analysis.

Cohort	Genotyping chip/s	Number of SNPs after filtering	Informative nuclear families		Informative families		Families with >1 child	
			Number of duos	Number of families	Number of duos	Number of families	Number of duos	Number of families
Carlantino, Italy (CARL)	Illumina HumanHap300	289671	6	2	16	6	29	13
French Canadians (FC)	Affymetrix 6.0 (900K)	478093	107	29	130	38	197	72
Friuli Venezia Giulia, Italy (FVG)	Illumina HumanHap300	302661	18	5	39	14	83	37
Korcula, Croatia	Human370CNV	294148	0	0	2	1	16	8
Netherlands Twin Registry (NTR)	Affymetrix 6.0 (900K)	413268	303	98	356	115	735	305
ORCADES, Orkney, Scotland	Illumina HumanHap300, Omni	152398	49	15	74	25	171	75
Queensland Twin Registry (QTR370)	Illumina HumanCNV370v1_C, HumanCNV370-Quadv3_C, Human610-Quadv1_B	273926	897	241	986	260	1100	317
Queensland Twin Registry (QTR610)	Illumina Human660W-Quad_v1, Human610-Quadv1	451905	1289	405	1292	406	2820	1170
Queensland Twin Registry (QTRCoreExome)	Illumina HumanCoreExome-12v1-0_C	245153	234	75	243	78	557	235
Val Borbera, Italy (VB)	Illumina HumanHap300	310788	30	9	48	17	156	71
Vis, Croatia	Illumina HumanHap300	252205	0	0	4	2	20	10
Total			3061	924	3558	1073	6311	2484
Total used			2909	872	3168	953	5117	1986

Supplementary Table 2. Summary of number of crossovers called by NFTOOLS and duoHMM, by cohort. We observed that the number of crossovers called by duoHMM was somewhat higher than expected in some cohorts, relative to the deCODE 2002 genetic map [32] and Hussin *et al.* [8]. When we excluded double crossovers within *X* SNPs of each other, where *X* was the average number of SNPs per megabase for that cohort, the numbers were in line with expectation, and so we used these filtered calls for all analyses except those presented in Supplementary Table 3.

Cohort	Parental meiosis	Average number of SNPs within 1Mb	duoHMM, fully informative meioses, raw counts							
			N. duos	minimum	25th percentile	median	mean	75th percentile	maximum	standard deviation
CARL	Maternal	104	47	22	35	41	40.17	43.5	62	7.81
FC	Maternal	172	166	23	36.25	42	43.71	49	98	10.44
FVG	Maternal	109	92	24	36.75	41	41.20	46.25	60	7.82
KORCULA	Maternal	106	11	31	33.5	38	42.73	49	68	11.72
NTR	Maternal	148	426	23	40	46	46.25	52	80	9.13
ORCADES	Maternal	55	143	21	34	39	39.55	45	62	7.46
QTR370	Maternal	98	1766	21	40	46	46.22	52	82	8.99
QTR610	Maternal	162	1399	19	43	48	48.90	56	89	9.38
QTRCoreExome	Maternal	88	364	25	42	47	47.77	53	76	8.71
VB	Maternal	111	104	19	33	41	40.80	47	61	9.52
VIS	Maternal	91	17	27	35	41	41.29	46	58	8.72
CARL	Paternal	104	24	19	23	26	26.00	29	34	4.10
FC	Paternal	172	137	17	26	29	29.73	33	68	6.03
FVG	Paternal	109	50	17	23	26	26.12	29.75	36	4.57
KORCULA	Paternal	106	4	22	27.25	30	28.75	31.5	33	4.79
NTR	Paternal	148	361	15	27	30	30.44	34	48	5.67
ORCADES	Paternal	55	86	14	23	25	25.64	30	36	4.56
QTR370	Paternal	98	1306	16	27	31	31.42	35	52	5.85
QTR610	Paternal	162	1348	16	29	32	32.79	36.25	53	5.91
QTRCoreExome	Paternal	88	268	17	28	31	31.38	35	46	5.15
VB	Paternal	111	69	17	22	25	25.35	28	34	4.12
VIS	Paternal	91	12	19	22.75	24.5	25.50	26.75	35	5.07

Supplementary Table 2 continued

Cohort	Parental meiosis	duoHMM, fully informative meioses, filtered counts							
		N. duos	minimum	25th percentile	median	mean	75th percentile	maximum	standard deviation
CARL	Maternal	47	20	34	38	38.23	42	62	7.79
FC	Maternal	166	19	33	39	39.83	45	61	8.41
FVG	Maternal	92	22	36	41	40.26	45.25	58	7.50
KORCULA	Maternal	11	29	32.5	38	41.82	48.5	67	11.78
NTR	Maternal	426	20	36	41	41.25	46.75	70	8.17
ORCADES	Maternal	143	20	33	38	38.54	43.5	59	7.24
QTR370	Maternal	1766	19	34	39	39.70	45	66	7.75
QTR610	Maternal	1399	17	33	38	38.72	44	66	7.75
QTRCoreExome	Maternal	364	22	36	41	40.91	46.25	64	7.76
VB	Maternal	104	19	31	38	38.23	43.25	59	9.03
VIS	Maternal	17	26	35	41	40.35	45	58	8.80
CARL	Paternal	24	17	23	25	25.54	29	33	4.01
FC	Paternal	137	17	25	27	27.15	29	46	4.28
FVG	Paternal	50	16	23	26	25.78	29	36	4.55
KORCULA	Paternal	4	22	26.5	29.5	28.50	31.5	33	4.80
NTR	Paternal	361	13	23	26	25.97	29	37	4.46
ORCADES	Paternal	86	13	22.25	25	25.23	28.75	36	4.56
QTR370	Paternal	1306	10	22	26	25.40	29	40	4.60
QTR610	Paternal	1348	11	21	24	24.02	27	40	4.45
QTRCoreExome	Paternal	268	16	23.75	26	26.07	29	37	4.12
VB	Paternal	69	13	21	23	23.04	26	33	3.95
VIS	Paternal	12	17	22.75	24	24.58	25	35	5.02

Supplementary Table 2 continued

Cohort	Parental meiosis	NFTOOLS, meioses from informative nuclear families							
		N. duos	minimum	25th percentile	median	mean	75th percentile	maximum	standard deviation
CARL	Maternal	NA	NA	NA	NA	NA	NA	NA	NA
FC	Maternal	122	21	33.25	40	40.49	46	63	9.13
FVG	Maternal	NA	NA	NA	NA	NA	NA	NA	NA
KORCULA	Maternal	NA	NA	NA	NA	NA	NA	NA	NA
NTR	Maternal	308	21	38	42	43.32	49	68	8.42
ORCADES	Maternal	56	26	34	39.5	40.38	44	62	8.20
QTR370	Maternal	1159	22	35	40	41.15	46	69	8.40
QTR610	Maternal	1343	18	36	42	42.03	48	73	8.75
QTRCoreExome	Maternal	240	25	37	41	42.21	48	67	7.83
VB	Maternal	29	32	39	43	44.31	48	61	8.92
VIS	Maternal	NA	NA	NA	NA	NA	NA	NA	NA
CARL	Paternal	NA	NA	NA	NA	NA	NA	NA	NA
FC	Paternal	122	17	24.25	27	27.33	30	36	4.05
FVG	Paternal	NA	NA	NA	NA	NA	NA	NA	NA
KORCULA	Paternal	NA	NA	NA	NA	NA	NA	NA	NA
NTR	Paternal	308	13	24	26	26.65	30	38	4.48
ORCADES	Paternal	56	16	22	25	24.98	27.25	35	4.44
QTR370	Paternal	1159	12	23	26	26.13	29	41	4.48
QTR610	Paternal	1345	14	23	26	26.27	29	41	4.28
QTRCoreExome	Paternal	240	16	24	26	26.11	28	39	3.97
VB	Paternal	29	15	23	25	25.28	27	44	5.10
VIS	Paternal	NA	NA	NA	NA	NA	NA	NA	NA

Supplementary Table 3. Summary of posteriors for β_{age} from different Bayesian hierarchical models fitted to raw duoHMM data. As for Table 2 in the main text, but using the raw duoHMM counts, before the removal of double crossovers over short intervals.

Parent	Model	Interpretation	Dataset	2.50%	25%	50%	75%	97.50%	$Pr(\beta_{age} > 0)$
Maternal	Model 1	additive	raw data, fully informative meioses	0.0433	0.0816	0.1022	0.1232	0.1659	0.9995
	Model 2	multiplicative	raw data, fully informative meioses	1.0010	1.0018	1.0023	1.0028	1.0037	0.9999
	Model 2*	multiplicative	raw data, fully + partially informative meioses	1.0010	1.0017	1.0021	1.0025	1.0033	1.0000
Paternal	Model 1	additive	raw data, fully informative meioses	-0.0375	-0.0104	0.0051	0.0196	0.0480	0.5912
	Model 2	multiplicative	raw data, fully informative meioses	0.9987	0.9997	1.0001	1.0006	1.0016	0.5754
	Model 2*	multiplicative	raw data, fully + partially informative meioses	0.9990	0.9997	1.0001	1.0004	1.0010	0.5391

Supplementary Table 4. Proportion of crossovers overlapping hotspots, by cohort. We determined the percentage of crossovers resolved to within 30kb that overlapped HapMap II hotspots, for the duoHMM calls on all meioses. Using the method of Coop *et al.* [7], we then estimated the proportion of crossovers overlapping hotspots by chance, and the proportion actually occurring in hotspots after correcting for this (α).

Cohort	Number of crossovers	% crossovers <30kb	% crossovers overlapping hotspots	% crossovers overlapping hotspots by chance	α	α 95% lower bound	α 95% upper bound
**Maternal**							
CARL	1333	33.8%	82.2%	32.3%	73.7%	70.6%	76.7%
FC	3618	39.6%	80.6%	30.4%	72.2%	70.3%	74.0%
FVG	3574	35.4%	82.0%	31.5%	73.8%	71.9%	75.6%
KORCULA	1065	34.1%	82.7%	32.4%	74.4%	70.9%	77.7%
NTR	12302	32.1%	82.0%	31.3%	73.8%	72.8%	74.8%
ORCADES	3879	21.9%	84.5%	34.6%	76.4%	74.6%	78.0%
QTR370	27574	34.9%	81.6%	32.3%	72.8%	72.1%	73.4%
QTR610	56585	47.2%	78.7%	28.2%	70.3%	69.8%	70.7%
QTRCoreExome	9094	27.8%	82.5%	34.3%	73.4%	72.2%	74.5%
VB	6509	37.8%	82.4%	31.2%	74.4%	73.0%	75.7%
VIS	1184	30.7%	82.2%	31.8%	73.9%	70.6%	77.0%
**Paternal**							
CARL	531	34.7%	85.1%	34.3%	77.3%	72.4%	81.7%
FC	2058	38.5%	83.3%	31.6%	75.6%	73.2%	77.9%
FVG	1210	34.4%	86.6%	32.8%	80.1%	77.1%	82.8%
KORCULA	336	33.7%	84.8%	32.8%	77.4%	71.2%	82.8%
NTR	6037	31.1%	85.4%	32.8%	78.3%	77.0%	79.6%
ORCADES	1522	21.5%	87.1%	36.0%	79.9%	77.1%	82.4%
QTR370	13363	34.3%	84.4%	33.3%	76.7%	75.8%	77.6%
QTR610	34857	46.2%	83.2%	29.8%	76.1%	75.6%	76.7%
QTRCoreExome	4439	28.4%	84.9%	35.2%	76.6%	75.0%	78.2%
VB	2495	37.3%	84.8%	32.3%	77.5%	75.4%	79.5%
VIS	480	31.8%	86.9%	33.9%	80.2%	75.2%	84.5%

Supplementary Table 5. Results from linear mixed models of the effect of parental age on the number of crossovers. This table gives the estimated effect size, β_{age}, its standard error, and the associated two-sided p-value. Note that we did not analyse the meioses from informative nuclear families in CARL and FVG because there were fewer than 20 per cohort.

Cohort	Maternal								
	duoHMM data, all fully informative duos			duoHMM data, duos from informative nuclear families			NFTOOLS data, duos from informative nuclear families		
	estimated β_{age}	standard error	p-value	estimated β_{age}	standard error	p-value	estimated β_{age}	standard error	p-value
CARL	-0.145	0.286	0.618	NA	NA	NA	NA	NA	NA
FC	-0.259	0.133	0.055	-0.424	0.157	0.008	-0.430	0.164	0.010
FVG	0.118	0.151	0.439	NA	NA	NA	NA	NA	NA
NTR	-0.073	0.111	0.510	-0.149	0.129	0.250	-0.141	0.134	0.294
ORCADES	-0.023	0.137	0.866	-0.022	0.226	0.923	0.022	0.231	0.923
QTR370	0.093	0.044	0.033	0.016	0.057	0.774	0.032	0.059	0.582
QTR610	0.150	0.058	0.010	0.157	0.058	0.007	0.158	0.061	0.010
QTRCoreExome	0.283	0.112	0.012	0.262	0.134	0.053	0.234	0.135	0.085
VB	0.338	0.215	0.123	0.493	0.234	0.049	0.674	0.245	0.013

Cohort	Paternal								
	duoHMM data, all fully informative duos			duoHMM data, duos from informative nuclear families			NFTOOLS data, duos from informative nuclear families		
	estimated β_{age}	standard error	p-value	estimated β_{age}	standard error	p-value	estimated β_{age}	standard error	p-value
CARL	NA	NA	NA	NA	NA	NA	NA	NA	NA
FC	-0.094	0.064	0.147	-0.095	0.068	0.169	-0.074	0.064	0.250
FVG	-0.044	0.122	0.719	NA	NA	NA	NA	NA	NA
NTR	0.105	0.057	0.068	0.099	0.062	0.113	0.060	0.063	0.344
ORCADES	0.073	0.098	0.459	0.107	0.114	0.357	0.118	0.109	0.283
QTR370	0.015	0.029	0.616	0.027	0.031	0.382	0.009	0.029	0.750
QTR610	-0.061	0.028	0.028	-0.060	0.028	0.031	-0.055	0.027	0.045
QTRCoreExome	-0.039	0.063	0.530	-0.045	0.060	0.451	-0.027	0.054	0.619
VB	0.045	0.104	0.665	-0.157	0.142	0.282	-0.322	0.179	0.088

Supplementary Table 6. Results from a meta-analysis of the effect of parental age on the number of crossovers. We analysed the effect sizes shown in Supplementary Table 5 using a fixed-effects meta-analysis, weighting them by their inverse variances [27]. This table gives the estimated effect size, β_{age}, its standard error, and the associated two-sided p-value, as well as the test statistic and p-value from Cochran's Q test for heterogeneity.

Dataset	Method	estimated β_{age}	standard error	p-value	test statistic from Cochran's Q test for heterogeneity	p-value from Cochran's Q test for heterogeneity
Maternal	duoHMM data, all informative duos	0.090	0.029	0.002	15.796	0.045
	duoHMM data, informative nuclear families	0.060	0.035	0.089	21.278	0.002
	NFTOOLS data, informative nuclear families	0.071	0.037	0.055	21.862	0.001
Paternal	duoHMM data, all informative duos	-0.015	0.017	0.364	11.050	0.136
	duoHMM data, informative nuclear families	-0.017	0.018	0.327	11.584	0.072
	NFTOOLS data, informative nuclear families	-0.022	0.017	0.203	9.433	0.151

Supplementary Table 7. Comparison of results from Model 1 fitted to NFTOOLS *versus* duoHMM data. As for Table 2, but for meioses from informative nuclear families.

Parent	Method	Model	Dataset	Interpretation	2.50%	25%	50%	75%	97.50%	Pr($\beta_{age} > 0$)
Maternal	NFTOOLS	Model 1	meioses from informative nuclear families	additive	-0.0081	0.0389	0.0641	0.0884	0.1363	0.9597
	duoHMM	Model 1	meioses from informative nuclear families	additive	-0.0283	0.0243	0.0513	0.0774	0.1228	0.8990
Paternal	NFTOOLS	Model 1	meioses from informative nuclear families	additive	-0.0543	-0.0333	-0.0210	-0.0096	0.0121	0.1079
	duoHMM	Model 1	meioses from informative nuclear families	additive	-0.0540	-0.0295	-0.0172	-0.0049	0.0187	0.1729

Supplementary Note 1

Funding Acknowledgements

PD is funded by a Wellcome Trust Senior Investigator Award (095552/Z/11/Z), and we also acknowledge funding from a Wellcome Trust Enhancement Grant (090532/Z/09/A) and a Wellcome Trust Core Award (090532/Z/09/Z). J.G.H. is a Human Frontiers Postdoctoral Fellow (LT-001017/2013-L).

Support for the Queensland Twin Registry cohort came from grants from the National Institutes of Health (AA07535, AA07728, AA13320, AA13321, AA14041, AA11998, AA17688, DA012854, DA019951), the Australian National Health and Medical Research Council (241944, 339462, 389927, 389875, 389891, 389892, 389938, 442915, 442981, 496739, 552485, 552498), the 5th Framework Programme (FP-5) GenomEUtwin Project (QLG2-CT-2002-01254) and the National Health and Medical Research Institute (Medical Bioinformatics Genomics Proteomics Program, 389891).

Support for the Netherlands Twin Register (NTR) studies and research was obtained from multiple grants from the Netherlands Organization for Scientific Research (NWO) and The Netherlands Organisation for Health Research and Development (ZonMW), the European Research Council (ERC-230374), Rutgers University Cell and DNA Repository (NIMH U24 MH068457-06), the Avera Institute, Sioux Falls, South Dakota (USA) and the National Institutes of Health (NIH R01 HD042157-01A1). Part of the NTR genotyping was funded by the Genetic Association Information Network (GAIN) of the Foundation for the National Institutes of Health and Grand Opportunity grants 1RC2 MH089951 and 1RC2 MH089995). We acknowledge support from VU University's Institute for Health and Care Research (EMGO+), the Neuroscience Campus Amsterdam (NCA) and the faculty of Psychology and Education of VU University.

The ORCADES cohort was supported by the Chief Scientist Office of the Scottish Government, the Royal Society, the MRC Human Genetics Unit, Arthritis Research UK and the European Union framework program 6 EUROSPAN project (contract no. LSHG-CT-2006-018947).

The Vis and Korcula studies were funded by grants from the Medical Research Council (UK) and from the Republic of Croatia Ministry of Science, Education and Sports (10810803150302). Korcula genotyping was funded by the European Union Framework Program 6 Project EUROSPAN (LSHGCT2006018947).

Research on the Val Borbera cohort was supported by funds from the following institutions: the Compagnia di San Paolo, Torino, Italy; Fondazione Cariplo, Italy; Telethon Italy; Ministry of Health, Ricerca Finalizzata 2007 and 2011-2012 and Public Health Genomics (CCM) Project 2010; PRIN 2009.

NC's research is supported by the Wellcome Trust (Grant Codes WT098051 and WT091310), the EU FP7 (EPIGENESYS Grant Code 257082 and BLUEPRINT Grant Code HEALTH-F5-2011-282510).
